# Detailed quantification of cardiac ventricular myocardial architecture in the embryonic and fetal mouse heart by application of structure tensor analysis to high resolution episcopic microscopic data

**DOI:** 10.3389/fcell.2022.1000684

**Published:** 2022-11-16

**Authors:** Patricia Garcia-Canadilla, Timothy J. Mohun, Bart Bijnens, Andrew C. Cook

**Affiliations:** ^1^ BCNatal—Barcelona Center for Maternal-Fetal and Neonatal Medicine (Hospital Sant Joan de Déu and Hospital Clínic, University of Barcelona), Barcelona, Spain; ^2^ Cardiovascular Diseases and Child Development, Institut de Recerca Sant Joan de Déu, Esplugues de Llobregat, Spain; ^3^ UCL Institute of Cardiovascular Science, London, United Kingdom; ^4^ The Francis Crick Institute, London, United Kingdom; ^5^ Institut d’Investigacions Biomèdiques August Pi i Sunyer (IDIBAPS), Barcelona, Spain; ^6^ ICREA, Barcelona, Spain

**Keywords:** myocyte orientation, myocardial development, high resolution episcopic microscopy, structure tensor analysis, helical angle (HA), intrusion angle

## Abstract

The mammalian heart, which is one of the first organs to form and function during embryogenesis, develops from a simple tube into a complex organ able to efficiently pump blood towards the rest of the body. The progressive growth of the compact myocardium during embryonic development is accompanied by changes in its structural complexity and organisation. However, how myocardial myoarchitecture develops during embryogenesis remain poorly understood. To date, analysis of heart development has focused mainly on qualitative descriptions using selected 2D histological sections. High resolution episcopic microscopy (HREM) is a novel microscopic imaging technique that enables to obtain high-resolution three-dimensional images of the heart and perform detailed quantitative analyses of heart development. In this work, we performed a detailed characterization of the development of myocardial architecture in wildtype mice, from E14.5 to E18.5, by means of structure tensor analysis applied to HREM images of the heart. Our results shows that even at E14.5, myocytes are already aligned, showing a gradual change in their helical angle from positive angulation in the endocardium towards negative angulation in the epicardium. Moreover, there is gradual increase in the degree of myocardial organisation concomitant with myocardial growth. However, the development of the myoarchitecture is heterogeneous showing regional differences between ventricles, ventricular walls as well as between myocardial layers, with different growth patterning between the endocardium and epicardium. We also found that the percentage of circumferentially arranged myocytes within the LV significantly increases with gestational age. Finally, we found that fractional anisotropy (FA) within the LV gradually increases with gestational age, while the FA within RV remains unchanged.

## 1 Introduction

The mammalian heart, which is one of the first organs to form and function during embryogenesis, develops from a simple tube into a complex organ with four chambers and four valves able to efficiently pump blood towards the rest of the body. One of the animal models to investigate the developing heart and related pathologies is the mouse embryo. During embryonic development, there is concomitant development of myocardial architecture and organisation. At early developmental stages, soon after looping, ventricular trabeculations start to develop which, apart from enhancing contractility, are also important in coordinating intraventricular conduction and increase myocardial oxygenation prior to septation and establishment of coronary circulation (Sedmera et al., 2000). With the completion of ventricular septation (E14.5), all definitive major cardiac structures are identifiable, and the outer layer of compact myocardium starts to expand (Captur et al., 2016; Paun et al., 2018). This process coincides with the invasion of the developing coronary vasculature from the epicardium (Sedmera et al., 2000), which enables continued perfusion of the myocardium, leading to further growth of the myocardial wall.

The progressive expansion/growth of the compact myocardium is accompanied by changes in its structural complexity and organisation. In the adult heart, myocytes are organised and aligned following a predominant (longitudinal) direction within the ventricular walls, and this predominant arrangement changes with myocardial depth, forming a 3-dimensional (3D) network (myocardial architecture or myoarchitecture). However, how myocardial architecture develops during embryogenesis is poorly understood. Most studies on myocardial development have used histology to characterise myocardial structural development (Sedmera et al., 1998; Sedmera et al., 2000; Wessels and Sedmera, 2004; Savolainen et al., 2009). However, this technique is very time consuming and only 2D images from tissue sections can be obtained, hindering the detailed quantification of the 3D arrangement of myocytes. Other authors have used diffusion tensor magnetic resonance imaging (DT-MRI) to describe the development of myocyte aggregates orientation in lambs and sheep (Abdullah et al., 2016; Le et al., 2021) and in humans (Mekkaoui et al., 2013; Pervolaraki et al., 2013; Nishitani et al., 2020). However, the resolution of DT-MRI is low (in the order of millimeters) which is not enough to quantify in detail the 3D arrangement of myocytes in tiny developing mouse hearts.

High resolution episcopic microscopy (HREM) is a novel microscopic imaging technique that enables to obtain high-resolution (in the order of microns) 3D images of whole organs, such as the heart (Mohun and Weninger, 2011), or embryos (Weninger et al., 2006; Mohun and Weninger, 2012a; Mohun and Weninger, 2012b). Paun et al. performed a detailed 3D quantification of the development of left ventricular myocardial complexity, using HREM images from mouse embryos and showed that regional complexity of the trabeculations increases longitudinally from base to apex and that overall complexity decreases with gestational age (GA) (Paun et al., 2018). However, a detailed characterisation of the development of myocardial architecture in mouse embryos has not yet been performed. We have recently shown that it is possible to use structure tensor-based methods to quantify the 3D arrangement of myocytes in HREM images from a mouse model of hypertrophy cardiomyopathy (Garcia-Canadilla et al., 2019).

In the present study, we aimed to perform a detailed quantification of embryonic development of myoarchitecture in wildtype mice, from E14.5 to E18.5, by means of structure tensor analysis applied to HREM images of the heart.

## 2 Materials and methods

### 2.1 Data samples

All specimens were handled in compliance with the Guide for the Care and Use of Laboratory Animals published by the US National Institutes of Health and with the approval of the MRC National Institute of Medical Research Ethical Review Panel. Mouse (*Mus musculus*) embryos were obtained from NIMR:Parkes (a robust outbred strain maintained at the MRC National Institute of Medical Research). For approximate embryo staging, detection of a vaginal plug was taken as gestation day 0.5 (GA0.5).

The detailed sample preparation procedure for HREM imaging has been described elsewhere (Weninger et al., 2006; Mohun and Weninger, 2011; Mohun and Weninger, 2012a; Mohun and Weninger, 2012b; Paun et al., 2018). Briefly, harvested embryos were agitated in phosphate buffered saline (PBS) solution to minimize retention of blood in embryonic heart. Potassium chloride was then added to ensure that hearts were arrested in diastole. Hearts were then isolated, washed in PBS and fixed in 4% paraformaldehyde. To remove remaining blood within the heart chambers, samples were then washed in repeated changes of distilled water for 30–60 min at room temperature. After overnight fixation in 4% paraformaldehyde (4°C), hearts were embedded in methacrylate resin (Mohun and Weninger, 2012a; Mohun and Weninger, 2012b). Samples were similarly positioned during embedding to ensure relatively reproducible base-to-apex sectioning during the HREM imaging process. A total of 66 embryo hearts with ages from E14.5 to E18.5 were included (E14.5: 14, E15.5: 14, E16.5: 12, E17.5: 12 and E18.5: 14; see Tables 1–5).

**TABLE 1:** Percentage of circumferentially (−20° < HA < 20°), longitudinal positive (HA > 20°) and longitudinal negative (HA < −20°) arranged myocytes within the left (LV) and right ventricle (RV), expressed as mean ± standard deviation.

N° samples	E14.5	E15.5	E16.5	E17.5	E18.5	*p*-value
	14	14	12	12	14	
Percentage of circumferentially arranged myocytes in the LV (%)						
Apical	27.1 ± 5.9^a^	31.1 ± 5.7^ab^	33.6 ± 4.3^b^	32.4 ± 4.1^b^	36.0 ± 3.1^b^	<0.001
Mid	33.4 ± 6.0^a^	36.4 ± 6.7^abc^	39.6 ± 4.0^bc^	36.0 ± 5.0^ab^	41.9 ± 3.1^c^	<0.001
Basal	26.3 ± 5.9^a^	29.2 ± 4.4^ab^	32.9 ± 3.7^b^	29.1 ± 4.5^ab^	33.7 ± 2.7^b^	<0.001
Percentage of circumferentially arranged myocytes in the RV (%)						
Apical	31.3 ± 5.9^a^	31.3 ± 5.0^a^	31.3 ± 4.4^a^	31.1 ± 5.4^a^	36.3 ± 5.1^a^	0.043
Mid	34.0 ± 5.7^a^	39.4 ± 4.6^b^	42.1 ± 3.3^bc^	39.0 ± 5.4^ab^	46.4 ± 4.6^c^	<0.001
Basal	30.6 ± 5.3^a^	32.6 ± 6.2^a^	39.0 ± 5.4^a^	29.2 ± 4.1^a^	32.3 ± 4.2^a^	0.123
Percentage of longitudinally positive arranged myocytes in the LV (%)						
Apical	40.3 ± 5.5^a^	39.6 ± 3.3^a^	37.7 ± 3.1^a^	37.9 ± 2.1^a^	37.2 ± 2.6^a^	0.118
Mid	35.8 ± 5.4^b^	33.6 ± 4.0^ab^	31.3 ± 2.9^a^	33.4 ± 3.4^ab^	30.8 ± 2.1^a^	0.008
Basal	37.3 ± 4.0^b^	33.8 ± 3.7^ab^	33.5 ± 3.8^ab^	34.2 ± 3.7^ab^	32.4 ± 2.4^a^	0.009
Percentage of longitudinally positive arranged myocytes in the RV (%)						
Apical	32.2 ± 8.1^a^	33.0 ± 2.9^a^	30.4 ± 4.7^a^	29.5 ± 2.6^a^	27.6 ± 3.9^a^	0.040
Mid	30.1 ± 6.4^b^	26.8 ± 3.2^ab^	25.3 ± 4.7^ab^	29.4 ± 4.8^ab^	24.7 ± 3.4^a^	0.010
Basal	32.9 ± 6.6^b^	30.7 ± 5.3^ba^	26.8 ± 3.4^a^	31.7 ± 3.5^ab^	29.4 ± 2.7^ab^	0.018
Percentage of longitudinally negative arranged myocytes in the LV (%)						
Apical	32.5 ± 4.7^b^	29.3 ± 3.3^ab^	28.6 ± 2.6^a^	29.7 ± 3.0^ab^	26.7 ± 1.9^a^	<0.001
Mid	30.8 ± 3.9^a^	30.0 ± 3.9^a^	29.1 ± 1.9^a^	30.6 ± 3.6^a^	27.3 ± 2.2^a^	0.044
Basal	36.3 ± 3.2^ab^	37.0 ± 2.4^b^	33.6 ± 3.1^a^	36.7 ± 2.8^ab^	33.9 ± 3.1^ab^	0.006
Percentage of longitudinally negative arranged myocytes in the RV (%)						
Apical	36.4 ± 7.1^a^	35.7 ± 4.0^a^	38.3 ± 6.8^a^	39.4 ± 7.3^a^	36.1 ± 4.3^a^	0.469
Mid	35.9 ± 5.3^b^	33.8 ± 4.5^ab^	32.6 ± 4.8^ab^	31.5 ± 4.6^ab^	28.9 ± 4.8^a^	0.005
Basal	36.5 ± 3.9^a^	36.7 ± 2.5^a^	39.3 ± 3.8^a^	39.1 ± 2.9^a^	38.2 ± 2.4^a^	0.071

Superscripts represent non-significant differences between corresponding pairs, at *p* = 0.05 as analyzed by Tukey’s HSD.

### 2.2 Image acquisition

The prepared samples were then sectioned using the HREM protocol as described by Mohun and Weninger (2012a) (Weninger et al., 2006). Briefly, HREM uses block-face imaging to produce perfectly registered digital image stacks capturing the 3D architecture of the embryonic heart at high resolution. Resulting datasets comprise 1,000–2,000 short-axis images, produced by successive removal of 2 μm (E14.5–E16.5) or 3 μm (E17.5, E18.5) sections (base-to-apex direction).

### 2.3 Image processing

**FIGURE 1:**
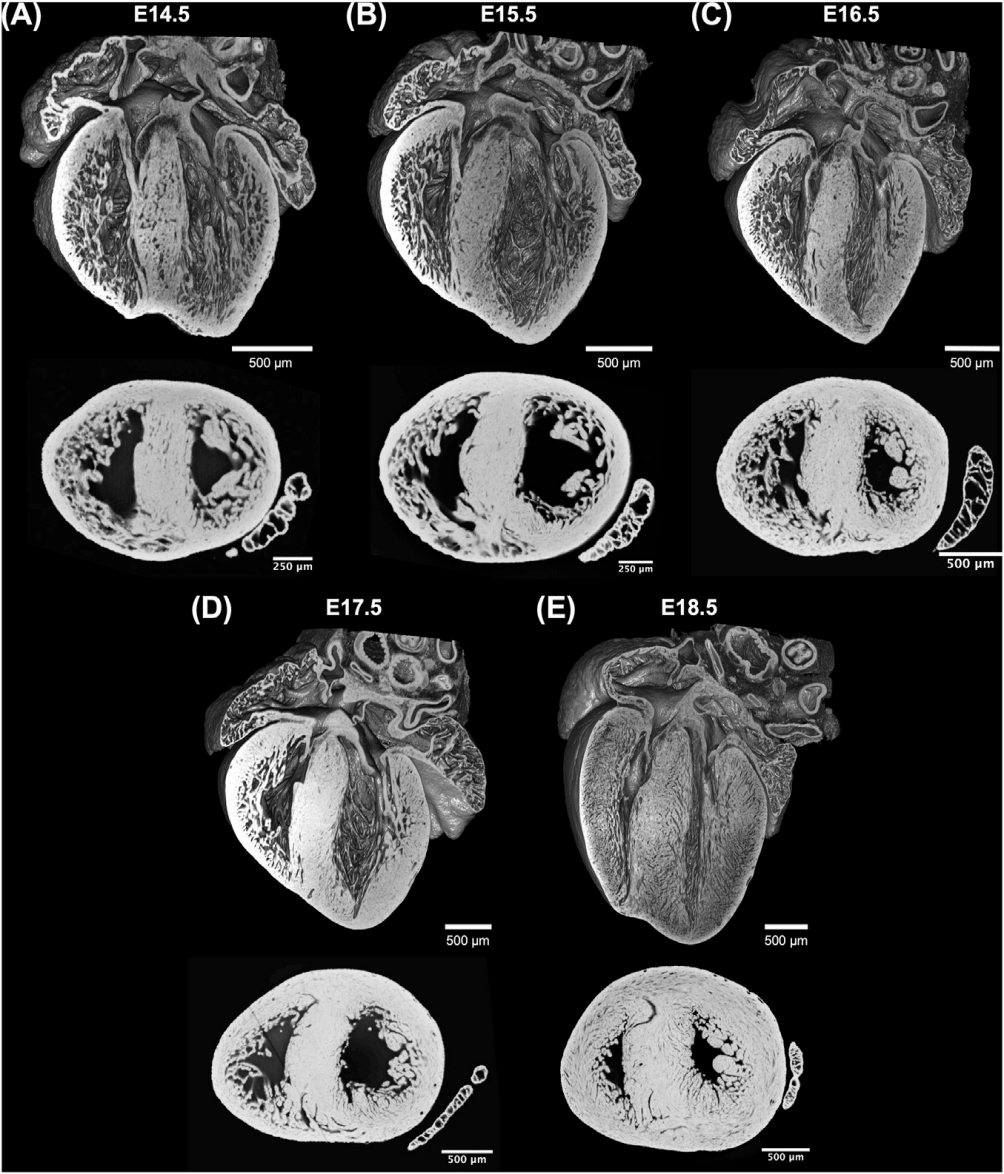
Whole-heart volume-rendered three-dimensional high-resolution episcopic microscopy reconstruction of the developing murine heart at **(A)** E14.5, **(B)** E15.5, **(C)** E16.5, **(D)** E17.5 and **(E)** E18.5 days, together with black/white inverted original episcopic microscopy 2D image.

Acquired HREM datasets were first isotropically resampled to obtain the same pixel size in all orthogonal planes, x, y and z, followed by automatic segmentation in Fiji (Schindelin et al., 2012). 3D volume-rendered reconstructions were performed in 3DSlicer (Fedorov et al., 2012). The volumetric visualizations of one dataset from each GA can be seen in Figure 1.

#### 2.3.1 Quantification of myocytes orientation

**FIGURE 2:**
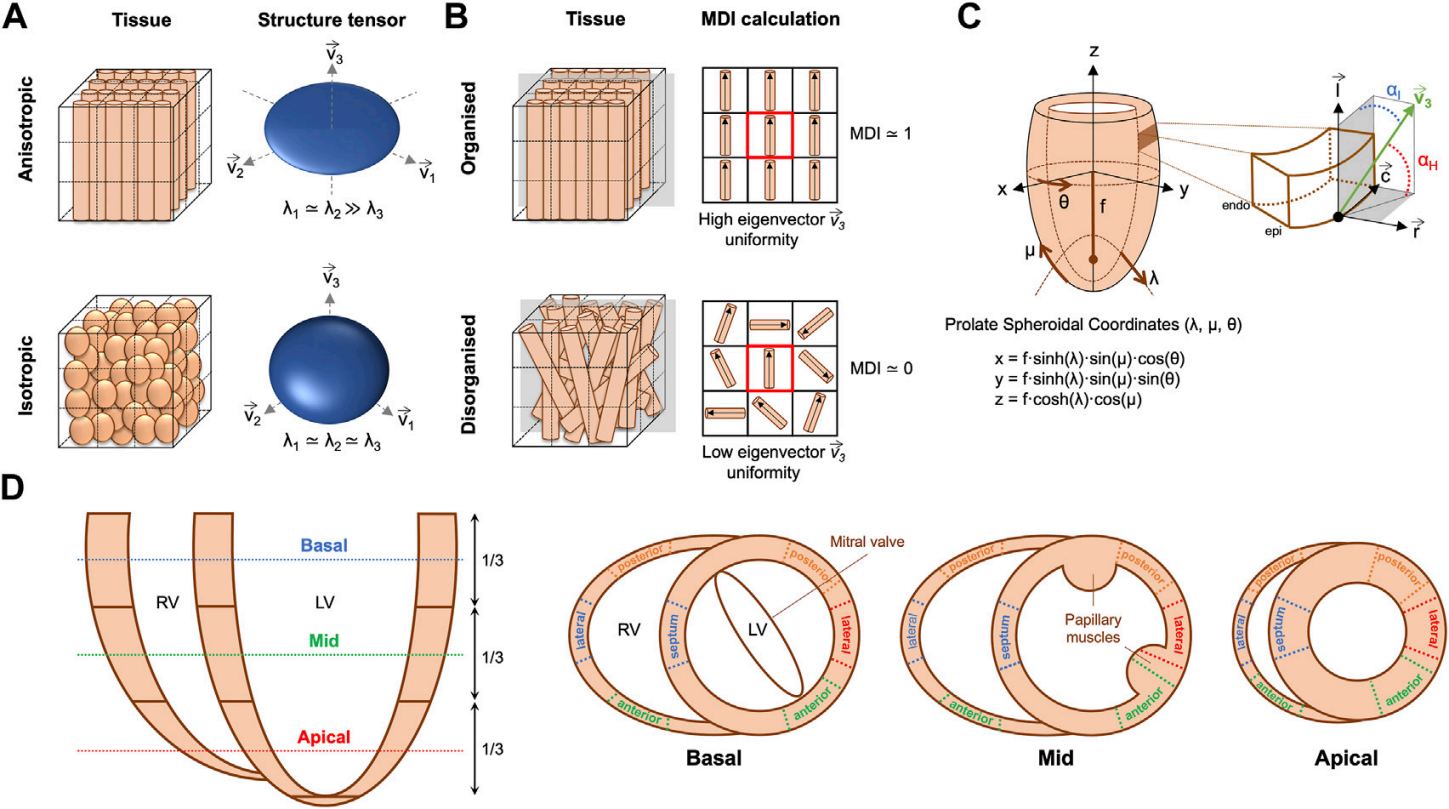
**(A)** Example of anisotropic vs. isotropic myocardium sub-volumes with their corresponding eigenvector system ( ${\vec{v}}_{i}$ , i = 1…3) and ellipsoids obtained with structure tensor analysis, showing high anisotropy represented by a flat ellipsoid when myocytes have a more elongated shape, vs. low anisotropy represented by a more spherical ellipsoid, when myocytes have a more spherical shape. **(B)** Schematic representation of the region of interest (ROI) defined to calculate the structure tensor in a given voxel, formed by the voxel itself (in the centre of the cube—red square in left panel) and their eight nearest voxels. In this drawing, each cylinder represents a single cardiomyocyte. The right panel illustrates the calculation of the myocardial disarray index (MDI) in the same ROI used to calculate the structure tensor. In this drawing, we have plotted the tertiary eigenvectors of the 2D plane indicated in grey in the right panel. MDI in a given voxel quantifies the angular uniformity between the tertiary eigenvectors of the voxel itself (red square) and the tertiary eigenvectors of their nearest neighbours. In an organised myocardium, all the eigenvectors have similar orientations and therefore MDI is close to 1. However, when myocardium is disorganized, all eigenvectors within the ROI have very different orientations, and therefore MDI is close to 0. **(C)** Scheme defining local prolate spheroidal coordinates of the LV, with transmural (*λ*), longitudinal (*μ*) and circumferential (θ) axis, and the helical (α_H_) and intrusion (α_I_) angles used to describe myocyte orientation. α_H_, is defined as the angle between the local short-axis or transverse plane and the tertiary eigenvector ${\vec{v}}_{3}$ . α_I_ is the angle between the local epicardial tangential plane and the tertiary eigenvector ${\vec{v}}_{3}$ . **(D)** Schematic representation of the 2D images slices and LV and RV segments selected to perform the quantification of myocyte aggregates orientation.

An in-house structure tensor-based method implemented in MATLAB (The MathWorks Inc., Natick, MA, United States, R2018a) (Baličević et al., 2015; Garcia-Canadilla et al., 2018; Garcia-Canadilla et al., 2019) was used to assess myocyte aggregates orientation. Briefly, for each image voxel, the gradient in the three directions was obtained using a central difference algorithm. The structure tensor was calculated as the cross product of gradient vectors. Eigen-decomposition was then applied to obtain the three eigenvectors and their eigenvalues. The eigenvector ( ${\vec{v}}_{3}$ ) with the smallest eigenvalue (λ_3_) was considered as the vector following the orientation of the myocyte aggregates in their longitudinal axis as it corresponded with the lowest intensity variation (see Figure 2A).

The orientation of ventricular myocyte aggregates was assessed by means of two angles: the helical angle (HA) and the intrusion angle (IA). To do that, we performed first a change of coordinate system from Cartesian to prolate spheroidal coordinates (Figure 2C). The prolate spheroidal coordinate system has the advantage of been physiologically meaningful with respect to the ellipsoidal shape of the left ventricle (LV) (Toussaint et al., 2013; Garcia-Canadilla et al., 2019). To do that, we first calculated the semi-foci distance *f* for each heart as follows: $=\sqrt{R_{a}^{2}-R_{b}^{2}}$ , where *R*_*a*_ and *R*_*b*_ are the major and minor axis of the LV ellipsoid respectively.

Then, the HA was calculated as the angle between the tertiary eigenvector ${\vec{v}}_{3}$ , and the local circumferential plane defined by the local radial ( $\vec{r}$ ) and circumferential ( $\vec{c}$ ) directions (Figure 2C). Whereas the IA was calculated as the angle between the tertiary eigenvector ${\vec{v}}_{3}$ , and the local epicardial tangential plane, defined by the local longitudinal ( $\vec{l}$ ) and circumferential ( $\vec{c}$ ) directions (Figure 2C) (Garcia-Canadilla et al., 2019).

Distributions of HA and IA within the LV and right ventricle (RV) were computed at three different apico-basal levels (Figure 2D). Then, the percentage of circumferentially, longitudinally positive and longitudinally negative arranged myocytes was estimated as the proportion of myocyte aggregates with a |HA| ≤ 20°, HA > 20° and HA < −20° respectively. Similarly, the percentage of myocyte aggregates with a |IA| ≤ 15°, 15° < |IA| ≤ 45° and |IA| > 45° was also computed. Mean values of the proportions of myocytes aggregates with different HA and IA within the LV (excluding the septum), septum and RV were plotted as a function of GA for the three apical-basal image slices and a linear regression fitting was performed. Additionally, distributions of HA and IA were also computed in each LV and RV wall (Figure 2D), to obtain regional information on HA and IA distribution.

Transmural profiles, from endo- to epicardium (or right-side endocardium in the case of the septum) of HA and IA were obtained for each LV and RV wall as illustrated in Figure 2D. Next, a linear regression fitting, *y = β*_*1*_ *·x + β*_*0*_, was applied to transmural HA and IA profiles to characterise their linearity. Linearity coefficient (*R* ^*2*^) and gradient (*β*_*1*_) over the normalised myocardial wall (0: endocardium—1: epicardium) in degrees (°) were computed. Trabeculations were manually removed and therefore not considered in all the different quantitative analyses.

#### 2.3.2 Quantification of myocardial ventricular organisation

In order to quantify the organisation of ventricular myocardium, two different parameters were computed: the fractional anisotropy (FA) (O’Donnell and Westin, 2011; Novo Matos et al., 2020) and the myocardial disarray index (MDI) (Garcia-Canadilla et al., 2019).

FA is a scalar value between 0 and 1 that describes the degree of anisotropy of the image gradient, similarly to DT-MRI. A value of 0 means that image gradient is isotropic with no marked direction, while a value close to 1 means that the image gradient is predominantly aligned in one direction (Figure 2A). FA was calculated from the eigenvalues (*λ*_*1*_, *λ*_*2*_, *λ*_*3*_) of the structure tensor as follows (O’Donnell and Westin, 2011; Novo Matos et al., 2020):

$$FA=\sqrt{\frac{3}{2}}\frac{\sqrt{{\left(\lambda_{1}-\hat{\lambda }\right)}^{2}+{\left(\lambda_{2}-\hat{\lambda }\right)}^{2}+{\left(\lambda_{3}-\hat{\lambda }\right)}^{2}}}{\sqrt{\lambda_{1}^{2}+\lambda_{2}^{2}+\lambda_{3}^{2}}}$$

where $\hat{\lambda }=\left(\lambda_{1}+\lambda_{2}+\lambda_{3}\right)/3$ being the mean value of eigenvalues.

To quantify myoarchitectural disarray we computed the MDI as previously done in similar HREM datasets (Garcia-Canadilla et al., 2019). Briefly, this index quantifies, for each image voxel, the uniformity of the myocytes’ longitudinal direction (represented by the tertiary eigenvector ${\vec{v}}_{3}$ , Figure 2B) within a neighbourhood that consists of the voxel itself and its nearest neighbours. For this study, we have defined a neighbourhood of 11 × 11 × 11 voxels to compute the MDI, which represents a subvolume of cardiac tissue of 22 µm^3^ × 22 µm^3^ × 22 µm^3^ for E14.5–E16.5 and of 33 µm^3^ × 33 µm^3^ × 33 µm^3^ for E17.5–E18.5 datasets. A large value of MDI at a given voxel indicates that the disarray is insignificant (all the myocytes within the neighbourhood have similar orientations), whereas smaller values of MDI denote a great loss of myocytes organisation or, in other words, high degree of myoarchitectural disarray (all the myocytes within the neighbourhood have very different orientations, Figure 2B).

Distributions and mean values of FA and MDI were obtained in the LV and RV as well as in each LV segment recapitulating the American Heart Association 17-segment cardiac model (apical segment 17 not analysed). Mean FA and MDI values within the LV (excluding the septum), septum and RV were plotted as a function of GA for the three apical-basal image slices (Figure 2D) and linear regression performed.

#### 2.3.3 3D vector representation and tracking of myocytes

3D plots of the longitudinal direction of the myocyte aggregates, represented by the tertiary eigenvector ${\vec{v}}_{3}$ , in mid LV short-axis slices were performed in Paraview (Ahrens et al., 2005) using a vector Glyph filter. 3D vectors were displayed after applying a tube filter and color-coded by HA.

### 2.4 Statistical analysis

Continuous variables were expressed as mean ± standard deviation or median [range] based on a normal distribution by Kolmogorov-Smirnov testing. Differences between groups (time points and/or ventricular chambers/walls) were analysed for statistical significance using one-way ANOVA followed by Tukey honestly significant difference (HSD) test for all pair-wise comparisons. A *p*-value < 0.05 indicated statistical significance. Statistical analysis was performed using SPSS (v28).

## 3 Results

### 3.1 Overall cardiac morphology

Three-dimensional reconstruction showed the detailed anatomy of the embryonic mouse hearts at different stages of development, including four chambers, atrioventricular valves and pectinate muscles (Figure 1). The increase in cardiac size with GA is also visible. Unlike the adult heart, in which the LV wall is thicker than the RV, in murine fetal hearts both ventricles have similar thickness. Also, the increase in thickness of the compact myocardium with gestational age, especially in the LV, is also evident.

### 3.2 Changes in myocardial architecture during fetal development

#### 3.2.1 Myocyte aggregates orientation

##### 3.2.1.1 Helical angle

**FIGURE 3:**
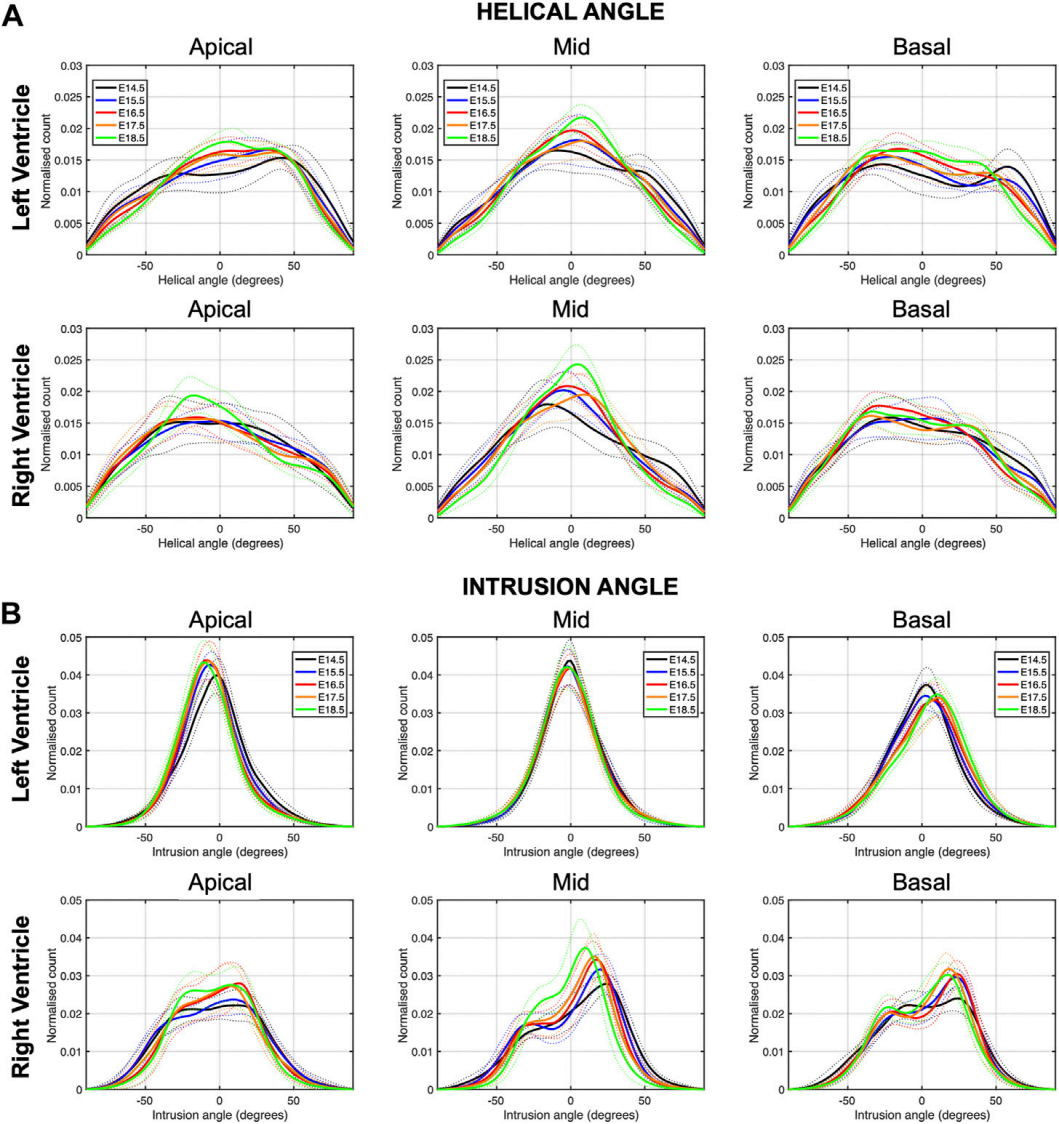
Histograms of **(A)** helical (HA) and **(B)** intrusion angle (IA) across the apical, mid-ventricular and basal LV and RV of murine hearts at E14.5, E15.5, E16.5, E17.5 and E18.5 days of development. Solid lines: age-group mean. Dotted lines: ± standard deviation.

There is an increase in the percentage of circumferentially arranged myocytes within the LV with developmental age, in particular from E14.5 to E16.5 (Apical: 27.1 ± 5.9% vs. 33.6 ± 4.3%, *p* = 0.009) (Table 1; Figure 3A; Supplementary Figure S1A), at the expense of a decrease in the percentage of longitudinally positive and longitudinally negative arranged myocytes (mid-myocardium E14.5 vs. E18.5: 35.8 ± 5.4% vs. 30.8 ± 2.1%, *p* = 0.007). In the RV, the percentage of circumferentially arranged myocytes also increases during the entire gestational period studied, in the mid and basal myocardium (mid: E14.5 vs. E18.5: 34.0 ± 5.7% vs. 46.4 ± 4.6%, *p* < 0.001) (Table 1; Figure 3A; Supplementary Figure S1A), similarly to the LV. However, in the apical part it remains unchanged up to E17.5, and then increases from E17.5 to E18.5. There are no significant differences in the amount of circumferentially arranged myocytes between the LV and RV. However, the septum has significant less circumferentially and more longitudinally positive arranged myocytes compared to the LV and RV during the whole gestation, especially in the mid and basal parts (Supplementary Figures S1A,B **)**. The percentage of longitudinally positive arranged myocytes within the LV and RV decreases with developmental age in particular in the apical and mid myocardium (Table 1; Supplementary Figure S1B). On the contrary, RV shows a significantly higher proportion of longitudinally negative arranged myocytes compared to the LV and septum, in the apical part during the whole gestation, and in the mid myocardium from E14.5 to E16.5 (Supplementary Figure S1C).

When looking at the distributions of HA in the different LV segments (Supplementary Figure S2), it is apparent that while anterior, posterior and lateral walls follow a unimodal distribution, the interventricular septum clearly shows a bimodal distribution, suggesting that septum belongs to both ventricles contributing to LV and RV function. Moreover, the increase in the percentage of circumferentially arranged myocytes with developmental age, is more evident in the posterior and lateral walls. Regarding the distributions of HA in the different RV segments, lateral wall shows predominantly longitudinally negative and circumferentially arranged myocytes, while anterior wall shows predominantly longitudinally negative myocytes and posterior wall longitudinally positive and circumferentially arranged myocytes at the base (Supplementary Figure S4A).

**FIGURE 4:**
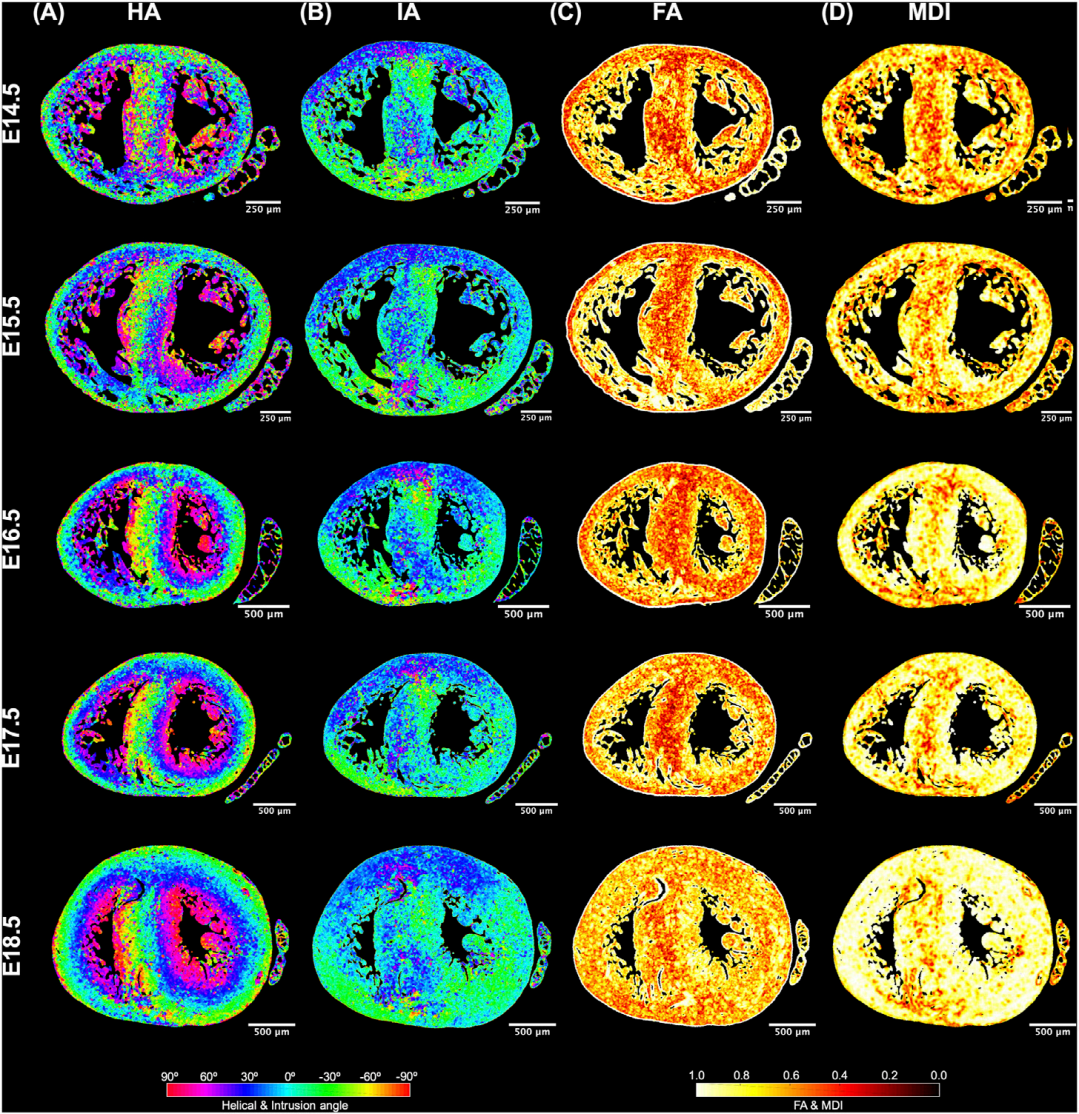
Quantification of myocyte aggregates orientation [*via* helical (HA) and intrusion angle (IA)] and myocyte aggregates organisation [*via* fractional anisotropy (FA) and myocardial disarray index (MDI)]. Local HA **(A)**, IA **(B)**, FA **(C)** and MDI **(D)** at mid-ventricular level, at E14.5, E15.5, E16.5, E17.5 and E18.5 days of development.

**FIGURE 5:**
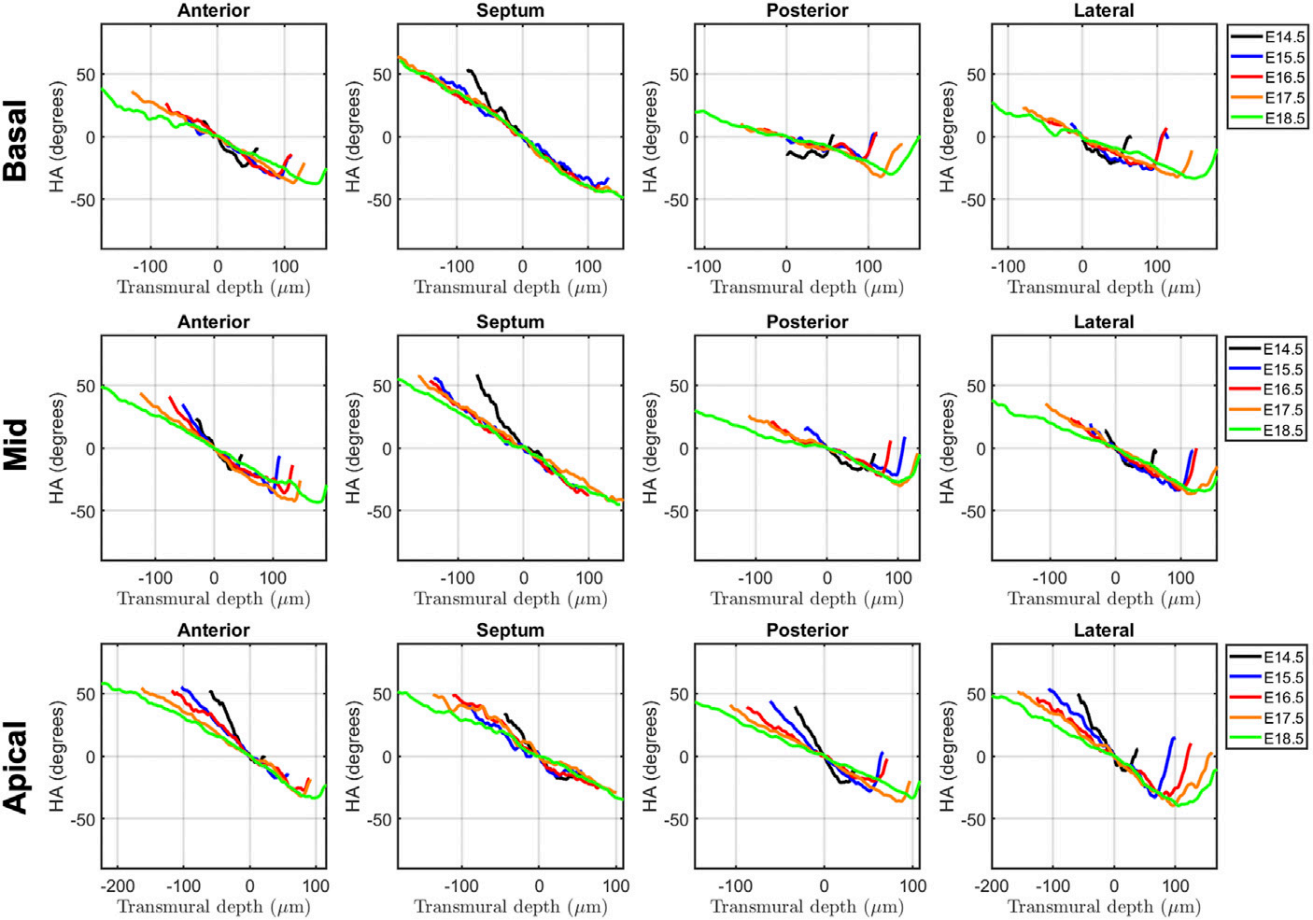
Left ventricular (LV) transmural profiles of helical angle (HA) across anterior, septal, posterior and lateral walls, from endocardium (negative transmural depth values) towards the epicardium (positive transmural depth values), at basal (top), mid (middle) and apical myocardium (bottom), at E14.5, E15.5, E16.5, E17.5 and E18.5 days of development. Solid lines represent age-group mean. Transmural depth of 0 µm represents the position where myocyte aggregates have a HA of 0°.

Regarding the change in HA along the myocardial wall, the cross-sectional views of the fetal myocardium at different developmental stages colour coded by HA, show a smooth and gradual change of HA in both LV and RV (Figure 4A), especially at late developmental stages. We have also plotted the transmural profile of HA along the different LV segments as a function of transmural depth from endo- to epicardium, where a transmural depth of 0 μm corresponds to the position of the mid-wall layer (HA = 0°) (Figure 5), as well as a function of the normalised transmural depth in the LV (Supplementary Figure S3A) and in the RV (Supplementary Figure S5A). The linearity of the transmural HA profiles significantly increases with growth in all the LV segments (Table 2; Figure 5; Supplementary Figure S3A) (mid lateral E14.5 vs. E18.5: 0.37 ± 0.22 and 0.94 ± 0.03 respectively, *p* < 0.001), while in the RV significantly increases in the basal and mid lateral and posterior walls (Table 3; Supplementary Figure S5A). There is also a significant increase in the slope of the HA transmural profile from E14.5 to E17.5 in all the LV segments (mid anterior E14.5 vs. E17.5: −39.95 ± 16.70° and −94.08 ± 19.10°, *p* < 0.001) (Table 2; Figure 5; Supplementary Figure S3A). We observed considerable local variability of the HA transmural profile when comparing the different LV segments. For example, in the mid and basal parts of the myocardium, the septal wall shows the steepest HA transmural profile (basal septum at E18.5: −119.54 ± 13.28°) while in the apical part, the LV lateral wall has the steepest profile (Table 2; Figure 5; Supplementary Figure S3A). We also noticed that the growth with developmental age in terms of HA, is different in the endo- than in the epicardium in the anterior, posterior and lateral LV walls, while in the septum, the growth seems to be similar towards both sides of the wall (Figure 5). Similar to the LV, there is also a significant increase in the slope of the HA transmural profile from E14.5 to E17.5 in all the RV segments except the apical lateral wall, with large variability across different segments as well (Table 3; Supplementary Figure S5A). Nevertheless, HA transmural profiles within the RV are less steep and linear compared to the LV, especially in the apical part.

**TABLE 2:** Parameters of the linear fitting: *y = β*_*1*_ *·x + β*_*0*_, of helical angle (HA) transmural profile within the left ventricle (LV), expressed as mean ± standard deviation.

N° samples	E14.5	E15.5	E16.5	E17.5	E18.5	*p*-value
	14	14	12	12	14	
Gradient (β_1_) of HA transmural profile (°)						
Basal anterior	−32.25 ± 17.61^a^	−46.88 ± 11.88^ab^	−59.15 ± 17.30^bc^	−76.53 ± 12.05^cd^	−71.84 ± 9.74^d^	<0.001
Basal septal	−81.06 ± 12.88^a^	−92.63 ± 16.64^ab^	−101.43 ± 17.27^b^	−126.19 ± 28.22^c^	−119.54 ± 13.28^c^	<0.001
Basal posterior	7.15 ± 17.96^a^	−8.30 ± 15.74^ab^	−20.10 ± 14.14^b^	−39.51 ± 14.35^c^	−43.77 ± 16.86^c^	<0.001
Basal lateral	0.45 ± 17.04^a^	−19.62 ± 12.51^b^	−36.67 ± 17.68^b^	−62.52 ± 14.24^c^	−57.77 ± 17.15^c^	<0.001
Mid anterior	−39.95 ± 16.70^a^	−59.53 ± 16.44^b^	−69.16 ± 16.40^b^	−94.08 ± 19.10^c^	−94.39 ± 11.83^c^	<0.001
Mid septal	−77.61 ± 14.36^a^	−85.60 ± 13.32^a^	−90.52 ± 12.59^ab^	−104.11 ± 20.27^b^	−104.30 ± 14.52^b^	<0.001
Mid posterior	−17.49 ± 10.81^a^	−30.72 ± 9.04^ab^	−36.79 ± 19.83^b^	−56.80 ± 17.18^c^	−54.82 ± 11.90^c^	<0.001
Mid lateral	−23.67 ± 20.11^a^	−42.47 ± 15.15^b^	−53.32 ± 18.25^b^	−75.84 ± 14.79^c^	−75.46 ± 8.20^c^	<0.001
Apical anterior	−65.23 ± 16.11^a^	−79.22 ± 14.59^ab^	−87.33 ± 16.55^bc^	−91.24 ± 12.23^bc^	−100.53 ± 8.64^c^	<0.001
Apical septal	−54.61 ± 17.63^a^	−66.21 ± 12.84^ab^	−84.07 ± 18.69^bc^	−84.87 ± 13.66^c^	−85.64 ± 20.09^c^	<0.001
Apical posterior	−63.97 ± 18.15^a^	−66.85 ± 14.80^ab^	−61.85 ± 15.66^a^	−81.50 ± 13.82^b^	−76.77 ± 10.29^ab^	0.004
Apical lateral	−58.37 ± 19.95^a^	−74.94 ± 12.37^ab^	−75.13 ± 22.91^abc^	−88.30 ± 14.72^bc^	−93.46 ± 11.57^c^	<0.001
Linearity (R^2^) of the linear fitting of HA transmural profile (unitless)						
Basal anterior	0.52 ± 0.29^a^	0.75 ± 0.15^b^	0.77 ± 0.15^b^	0.90 ± 0.07^b^	0.87 ± 0.08^b^	<0.001
Basal septal	0.88 ± 0.06^a^	0.90 ± 0.08^ab^	0.94 ± 0.04^b^	0.96 ± 0.02^b^	0.96 ± 0.01^b^	<0.001
Basal posterior	0.25 ± 0.23^a^	0.24 ± 0.25^a^	0.39 ± 0.26^ab^	0.66 ± 0.24^c^	0.66 ± 0.23^bc^	<0.001
Basal lateral	0.17 ± 0.15^a^	0.23 ± 0.21^a^	0.53 ± 0.23^b^	0.79 ± 0.14^c^	0.78 ± 0.23^c^	<0.001
Mid anterior	0.63 ± 0.22^a^	0.77 ± 0.11^b^	0.84 ± 0.05^bc^	0.95 ± 0.03^c^	0.96 ± 0.03^c^	<0.001
Mid septal	0.85 ± 0.09^a^	0.92 ± 0.05^b^	0.93 ± 0.04^b^	0.95 ± 0.04^b^	0.95 ± 0.03^b^	<0.001
Mid posterior	0.33 ± 0.23^a^	0.54 ± 0.15^b^	0.64 ± 0.27^bc^	0.83 ± 0.13^c^	0.81 ± 0.11^c^	<0.001
Mid lateral	0.37 ± 0.22^a^	0.61 ± 0.21^b^	0.74 ± 0.21^bc^	0.88 ± 0.08^cd^	0.94 ± 0.03^d^	<0.001
Apical anterior	0.80 ± 0.16^a^	0.90 ± 0.05^b^	0.91 ± 0.05^b^	0.93 ± 0.03^b^	0.95 ± 0.02^b^	<0.001
Apical septal	0.61 ± 0.23^a^	0.77 ± 0.12^b^	0.82 ± 0.12^b^	0.87 ± 0.05^b^	0.89 ± 0.12^b^	<0.001
Apical posterior	0.77 ± 0.19^a^	0.81 ± 0.06^a^	0.88 ± 0.07^ab^	0.95 ± 0.02^b^	0.96 ± 0.03^b^	<0.001
Apical lateral	0.68 ± 0.20^a^	0.69 ± 0.09^a^	0.76 ± 0.12^ab^	0.78 ± 0.09^ab^	0.86 ± 0.09^b^	0.002

Superscripts represent non-significant differences between corresponding pairs, at *p* = 0.05 as analyzed by Tukey’s HSD.

**TABLE 3:** Parameters of the linear fitting: *y = β*_*1*_ *·x + β*_*0*_, of helical angle (HA) transmural profile within the right ventricle (RV), expressed as mean ± standard deviation.

N° samples	E14.5	E15.5	E16.5	E17.5	E18.5	*p*-value
	14	14	12	12	14	
Gradient (β_1_) of HA transmural profile (°)						
Basal anterior	−11.19 ± 13.91^ab^	10.14 ± 33.99^a^	−18.66 ± 19.86^b^	−15.41 ± 26.20^ab^	−26.25 ± 27.29^b^	0.006
Basal lateral	−13.19 ± 17.02^a^	−37.27 ± 19.36^b^	−45.14 ± 13.86^bc^	−61.22 ± 13.03^cd^	−66.90 ± 21.58^d^	<0.001
Basal posterior	−41.41 ± 14.41^a^	−56.84 ± 18.09^ab^	−66.59 ± 10.24^bc^	−86.48 ± 16.58^cd^	−79.46 ± 11.84^d^	<0.001
Mid anterior	−17.53 ± 23.51^a^	−25.33 ± 21.26^ab^	−22.90 ± 13.27^ab^	−35.97 ± 19.83^ab^	−38.09 ± 12.41^ab^	0.027
Mid lateral	−17.40 ± 18.65^a^	−19.58 ± 17.47^a^	−26.97 ± 13.47^a^	−46.03 ± 15.88^b^	−46.95 ± 17.73^b^	<0.001
Mid posterior	−32.92 ± 15.30^a^	−48.45 ± 16.27^a^	−39.92 ± 24.61^ab^	−70.61 ± 17.94^bc^	−65.65 ± 12.48^c^	<0.001
Apical anterior	3.18 ± 15.66^a^	−6.43 ± 24.05^a^	1.69 ± 28.14^a^	−18.93 ± 21.68^a^	−20.12 ± 21.57^a^	0.019
Apical lateral	−7.87 ± 27.15^a^	−3.06 ± 23.57^a^	6.08 ± 18.51^a^	−9.34 ± 28.74^a^	−11.86 ± 27.30^a^	0.428
Apical posterior	−11.29 ± 15.82^a^	−36.95 ± 29.45^abc^	−20.77 ± 46.65^ab^	−54.89 ± 33.91^c^	−51.49 ± 14.97^bc^	<0.001
Linearity (R^2^) of the linear fitting of HA transmural profile (unitless)						
Basal anterior	0.29 ± 0.24^a^	0.47 ± 0.29^a^	0.40 ± 0.32^a^	0.52 ± 0.31^a^	0.59 ± 0.31^a^	0.102
Basal lateral	0.30 ± 0.24^a^	0.55 ± 0.26^b^	0.73 ± 0.14^bc^	0.83 ± 0.16^c^	0.87 ± 0.14^c^	<0.001
Basal posterior	0.71 ± 0.18^a^	0.81 ± 0.14^ab^	0.86 ± 0.08^b^	0.85 ± 0.06^b^	0.88 ± 0.06^b^	0.002
Mid anterior	0.52 ± 0.34^a^	0.60 ± 0.27^a^	0.61 ± 0.30^a^	0.73 ± 0.26^a^	0.75 ± 0.18^a^	0.184
Mid lateral	0.36 ± 0.32^a^	0.33 ± 0.22^a^	0.40 ± 0.23^ab^	0.55 ± 0.26^ab^	0.67 ± 0.23^b^	0.004
Mid posterior	0.64 ± 0.29^ab^	0.73 ± 0.22^ab^	0.63 ± 0.28^a^	0.86 ± 0.09^b^	0.87 ± 0.09^b^	0.006
Apical anterior	0.16 ± 0.17^a^	0.27 ± 0.23^a^	0.33 ± 0.29^a^	0.45 ± 0.32^a^	0.38 ± 0.31^a^	0.078
Apical lateral	0.19 ± 0.21^a^	0.20 ± 0.15^a^	0.11 ± 0.14^a^	0.15 ± 0.17^a^	0.21 ± 0.23^a^	0.648
Apical posterior	0.27 ± 0.26^a^	0.54 ± 0.29^ab^	0.50 ± 0.31^ab^	0.62 ± 0.35^b^	0.75 ± 0.20^b^	<0.001

Superscripts represent non-significant differences between corresponding pairs, at *p* = 0.05 as analyzed by Tukey’s HSD.

##### 3.2.1.2 Intrusion angle

Regarding the IA, the percentage of aggregated myocytes that deviate markedly from the tangential plane, with an |IA| > 45°, is always less than 10% in the LV during the whole gestation (Table 4; Figure 3B; Supplementary Figure S6C), while at early developmental stages, the RV has a significantly higher proportion of aggregated myocytes with an |IA| > 45°, but decreases with gestation, especially from E14.5 to E16.5 (Apical: 14.5 ± 6.4% vs. 7.3 ± 2.2%, *p* < 0.001), and remains below 10% the rest of gestation (Table 4; Figure 3B; Figure 4B; Supplementary Figure S6C). On the other hand, the percentage of aggregated myocytes with an |IA| ≤ 15° within the RV, increases with gestation, especially in the mid myocardium (from 31.1% at E14.5–46.4% at E18.5, *p* < 0.001) (Table 4; Figure 3B; Figure 4B; Supplementary Figure S6A). However, this proportion is always significantly lower in the RV and septum compared to LV, suggesting than RV and septum are less organised than the LV during fetal life. We also noted a change from prevalence of positive IA at the base to negative angles at the apex in the LV, from E16.5 onwards (Figure 3B), as have been previously described in the murine adult heart (Schmitt et al., 2009). When looking at the distribution of IA across the different LV and RV walls (Supplementary Figures S2B, S4B), the septum and anterior RV wall show the lowest proportion of aggregated myocytes with a |IA| ≤ 15°.

**TABLE 4:** Percentage of myocytes arranged parallel to the epicardial layer (|IA| < 15°), with an IA of 15° < |IA| < 45° and with an |IA| > 45° within the left (LV) and right ventricle (RV), quantified at apical, mid and basal levels, expressed as mean ± standard deviation.

N° samples	E14.5	E15.5	E16.5	E17.5	E18.5	*p*-value
	14	14	12	12	14	
Percentage of myocytes within the LV with an |IA| ≤ 15° (%)						
Apical	51.5 ± 4.4^a^	53.0 ± 4.1^a^	52.9 ± 5.0^a^	52.6 ± 2.9^a^	50.4 ± 4.2^a^	0.438
Mid	55.7 ± 5.3^a^	55.0 ± 4.7^a^	54.6 ± 4.0^a^	54.2 ± 4.7^a^	56.3 ± 4.5^a^	0.804
Basal	49.8 ± 5.2^a^	47.2 ± 4.0^ab^	43.8 ± 5.7^b^	43.1 ± 6.2^b^	43.4 ± 4.2^b^	0.003
Percentage of myocytes within the RV with an |IA| ≤ 15° (%)						
Apical	32.5 ± 4.6^a^	33.9 ± 4.5^ab^	40.5 ± 7.4^c^	40.6 ± 6.7^c^	40.1 ± 4.9^bc^	<0.001
Mid	31.1 ± 5.3^a^	31.7 ± 7.1^a^	37.1 ± 8.0^ab^	40.7 ± 4.7^bc^	46.4 ± 9.5^c^	<0.001
Basal	33.0 ± 4.7^a^	32.8 ± 4.8^a^	31.7 ± 4.0^a^	35.7 ± 5.3^a^	35.7 ± 5.3^a^	0.108
Percentage of myocytes within the LV with an 15° < |IA| ≤ 45° (%)						
Apical	41.9 ± 3.1^a^	42.2 ± 3.2^a^	42.2 ± 3.7^a^	42.5 ± 2.3^a^	44.8 ± 3.0^a^	0.101
Mid	39.9 ± 4.1^a^	40.7 ± 4.0^a^	40.1 ± 3.1^a^	40.0 ± 3.8^a^	38.7 ± 4.0^a^	0.732
Basal	43.7 ± 3.5^a^	45.8 ± 2.6^ab^	48.0 ± 3.3^b^	49.0 ± 4.1^b^	49.0 ± 3.2^b^	<0.001
Percentage of myocytes within the RV with an 15° < |IA| ≤ 45° (%)						
Apical	53.0 ± 4.2^a^	51.4 ± 3.3^a^	52.2 ± 5.9^a^	49.7 ± 3.9^a^	52.6 ± 2.7^a^	0.266
Mid	55.5 ± 2.9^ab^	58.4 ± 4.3^a^	55.8 ± 6.1^a^	52.9 ± 3.2^ab^	49.6 ± 8.5^b^	0.001
Basal	53.8 ± 3.9^a^	57.8 ± 3.9^ab^	59.3 ± 3.1^b^	56.0 ± 3.9^ab^	56.3 ± 4.3^ab^	0.008
Percentage of myocytes within the LV with an |IA| > 45° (%)						
Apical	6.6 ± 1.8^a^	4.8 ± 1.3^b^	4.9 ± 1.7^b^	4.9 ± 1.0 ^b^	4.8 ± 1.8^b^	0.009
Mid	4.3 ± 1.3^a^	4.3 ± 1.5^a^	5.2 ± 1.1^a^	5.7 ± 1.7^a^	5.0 ± 1.2^a^	0.038
Basal	6.4 ± 1.9^a^	7.0 ± 2.1^a^	8.3 ± 2.7^a^	8.0 ± 2.8^a^	7.7 ± 2.3^a^	0.288
Percentage of myocytes within the RV with an |IA| > 45° (%)						
Apical	14.5 ± 6.4^ab^	14.7 ± 4.8^a^	7.3 ± 2.2^c^	9.7 ± 4.3^bc^	7.4 ± 3.7^c^	<0.001
Mid	13.4 ± 4.0^a^	9.9 ± 3.8^b^	7.1 ± 2.2^bc^	6.4 ± 2.2^c^	4.0 ± 1.4^c^	<0.001
Basal	13.2 ± 4.1^a^	9.3 ± 2.5^b^	9.0 ± 2.6^b^	8.3 ± 2.1^b^	8.0 ± 2.6^b^	<0.001

Superscripts represent non-significant differences between corresponding pairs, at *p* = 0.05 as analyzed by Tukey’s HSD.

Regarding the change in IA along the myocardial wall, Supplementary Figure S3B shows the transmural course of IA in basal, mid-ventricular and apical slices of the LV anterior, septal, posterior and lateral walls, without significant changes in its course with development, except from the apical anterior wall. Similarly, Supplementary Figure S5B shows the transmural course of IA in basal, mid-ventricular and apical slices of the RV anterior, lateral and posterior walls, without significant changes in its course with development neither. Linearity (*R* ^*2*^) and gradient (*β*_*1*_) coefficients of the linear fitting of the IA transmural profiles for the different LV and RV segments are also provided in Supplementary Tables S1, S2, respectively.

#### 3.2.2 Myocardial ventricular organisation

**FIGURE 6:**
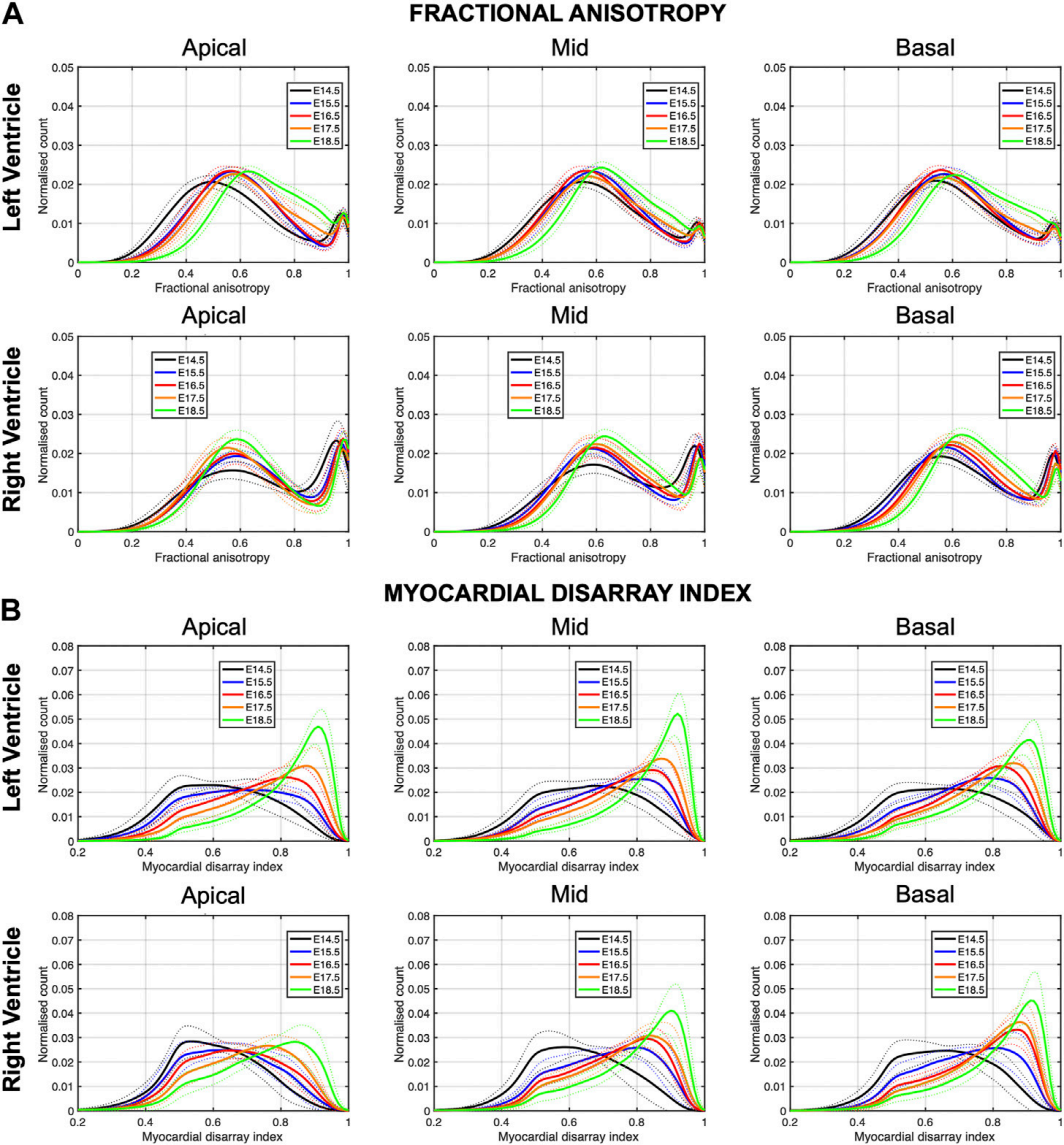
Histograms of **(A)** fractional anisotropy (FA) and **(B)** myocardial disarray index (MDI) across the apical, mid-ventricular and basal LV and RV of murine hearts at E14.5, E15.5, E16.5, E17.5 and E18.5 days of development. Solid lines: age-group mean. Dotted lines: ± standard deviation.

**FIGURE 7:**
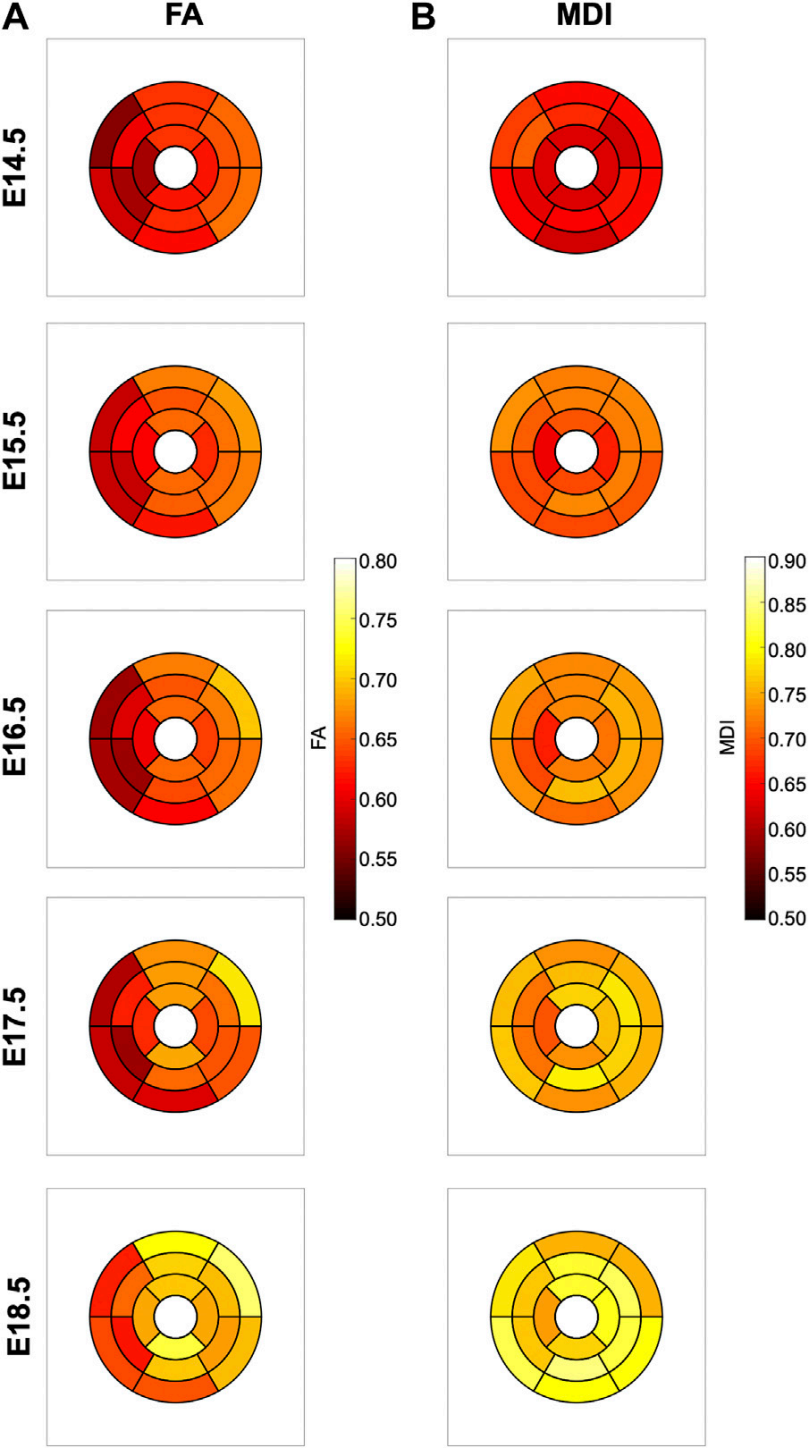
Seventeen-segment AHA bull’s eye plots quantifying fractional anisotropy (FA) **(A)** and myocardial disarray index (MDI) **(B)** in developing murine hearts at E14.5, E15.5, E16.5, E17.5 and E18.5 days of gestation.

In Figure 6, we have plotted the distributions of FA and MDI within the LV and RV, in the three apico-basal cross-sections for all gestational ages. The average FA and MDI values within the whole LV and RV cross-sections are provided in Table 5 and plotted as a function of GA in Supplementary Figure S7. Mean values of FA and MDI for each LV segment were also plotted in Figure 7.

**TABLE 5:** Fractional anisotropy (FA) and myocardial disarray index (MDI) in the left (LV) and right ventricle (RV), quantified at apical, mid and basal levels, expressed as mean ± standard deviation.

N° samples	E14.5	E15.5	E16.5	E17.5	E18.5	*p*-value
	14	14	12	12	14	
Fractional anisotropy						
Left ventricle						
Apical	0.55 ± 0.03^a^	0.60 ± 0.02^b^	0.60 ± 0.03^b^	0.63 ± 0.02^c^	0.68 ± 0.02^d^	<0.001
Mid	0.59 ± 0.03^a^	0.61 ± 0.03^ab^	0.59 ± 0.02^ab^	0.62 ± 0.03^b^	0.66 ± 0.02^c^	<0.001
Basal	0.58 ± 0.03^a^	0.60 ± 0.04^a^	0.59 ± 0.02^a^	0.61 ± 0.05^a^	0.67 ± 0.02^b^	<0.001
Right ventricle						
Apical	0.68 ± 0.06^ab^	0.66 ± 0.03^ab^	0.66 ± 0.06^ab^	0.63 ± 0.03^a^	0.65 ± 0.03^b^	0.058
Mid	0.67 ± 0.04^ab^	0.65 ± 0.02^a^	0.67 ± 0.05^ab^	0.67 ± 0.04^ab^	0.69 ± 0.02^b^	0.044
Basal	0.63 ± 0.03^a^	0.63 ± 0.02^ab^	0.65 ± 0.02^ab^	0.66 ± 0.04^bc^	0.68 ± 0.02^c^	<0.001
Myocardial disarray index						
Left ventricle						
Apical	0.62 ± 0.05^a^	0.67 ± 0.03^b^	0.74 ± 0.04^c^	0.79 ± 0.03^d^	0.84 ± 0.02^e^	<0.001
Mid	0.65 ± 0.67^a^	0.72 ± 0.04^b^	0.76 ± 0.04^bc^	0.79 ± 0.03^c^	0.85 ± 0.02^d^	<0.001
Basal	0.65 ± 0.05^a^	0.72 ± 0.04^b^	0.76 ± 0.03^bc^	0.78 ± 0.05^c^	0.83 ± 0.03^d^	<0.001
Right ventricle						
Apical	0.60 ± 0.04^a^	0.64 ± 0.05^ab^	0.66 ± 0.04^bc^	0.71 ± 0.04^cd^	0.75 ± 0.05^d^	<0.001
Mid	0.63 ± 0.06^a^	0.71 ± 0.05^b^	0.75 ± 0.03^bc^	0.77 ± 0.04^cd^	0.82 ± 0.04^d^	<0.001
Basal	0.65 ± 0.05^a^	0.73 ± 0.05^b^	0.78 ± 0.04^c^	0.80 ± 0.04^cd^	0.84 ± 0.03^d^	<0.001

Superscripts represent non-significant differences between corresponding pairs, at *p* = 0.05 as analyzed by Tukey’s HSD.

It can be observed that FA in the LV gradually increases throughout the development, particularly from E16.5 to E18.5, potentially due to changes in myocytes size and shape (Figures 4C, 7A; Supplementary Figure S7A). However, FA within the RV remains almost constant in the apical and mid myocardium, with a slight increase from E17.5 to E18.5, while it increases in the basal part, similarly to LV (Figure 4C; Supplementary Figure S5). According to bull’s eye plot of Figure 7A and Supplementary Figure S7A, FA in the septum is always significantly smaller than in the rest of ventricular walls.

**FIGURE 8:**
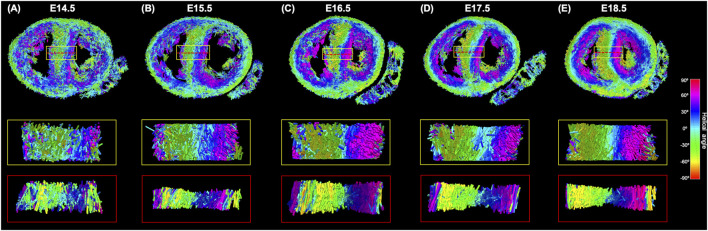
Three-dimensional representation of the tertiary eigenvector representing the longitudinal direction of aggregated myocytes for a single mid-LV slice of the developing murine heart at **(A)** E14.5, **(B)** E15.5, **(C)** E16.5, **(D)** E17.5 and **(E)** E18.5 days of gestation. The insets represent magnified areas within the septum, illustrating well how cardiomyocytes show different angles of intrusion throughout the septal wall, and how the myocardium becomes more organised with gestational age.

Regarding MDI, it increases during gestation as myocytes align and myocardium becomes more organised in all the myocardial walls (Figures 4D, 7B; Supplementary Figure S7B). This increase in the level of myocyte organisation with development can be also visualised in the 3D fibre tracts plot in Figure 8. From E16.5 onwards, we observed small foci of low myocyte organisation (voxels of MDI < 0.5 appearing as red/burgundy zones; Figure 4D) at the superior and inferior RV insertion points to the septum. From mid to late gestation, MDI is significantly higher in the LV than in the septum, in the apical and mid myocardium (Supplementary Figure S7B).

## 4 Discussion

In this study, we have shown quantitatively, for the first time, how myocardial architecture develops in the latter gestation mouse embryo and fetus. Our results show that, even at E14.5, around the time of completion of ventricular septation, myocytes are already aligned, following a predominant direction. This main direction changes gradually throughout the wall depth, from positive angulation in the endocardium towards negative angulation in the epicardium, and do not support a three-layered model of the myocardium (Torrent-Guasp et al., 2001). If a unique myocardial band would exist, as suggested by Guasp and colleagues, an abrupt change in HA would be expected. We have also shown that there is an increase in the linearity and slope of HA transmural profiles with gestational age (Figure 5; Supplementary Figure S2; Table 2). For instance, at E14.5, the HA in the mid LV anterior wall changes from about +20° to −20°, from endo to epicardium and its average linearity coefficient is 0.63, while at E18.5, the HA changes from about +40° to −40° with a linearity coefficient of 0.96 (Table 2). Nishitani et al. reported that transmural HA range, measured by DT-MRI in human fetal hearts, was almost constant between 80° and 120° and did not change with growth (Nishitani et al., 2020). However, the resolution of this technique, was 100–150 μm, which is insufficient to quantify detailed transmural HA change, especially in smaller samples (8 weeks of GA), as illustrated in their Figure 2. We have also found that the percentage of circumferentially arranged myocytes within the LV significantly increases with gestational age, at the expense of a reduction in the positive-angled myocytes, likely as a response to increased pressure loading, given the smallest radius of curvature, and thus lowest wall stress, in the circumferentially direction. Our results are consistent with previous DT-MRI studies in lambs (Abdullah et al., 2016), also showing an increase in the percentage of circumferentially arranged myocytes with gestational age. We have also noticed that ventricular myocardial growth is heterogeneous, showing different growth patterns between ventricles, between different ventricular regions (Figure 5; Supplementary Figure S3), as well as across the myocardial wall. This differential growth patterning between the endocardium and epicardium of the LV could be explained by the different embryonic origins and signalling pathways of the endocardium, myocardium and epicardium (Smith and Bader, 2007), as well as by the gradient of decreasing proliferation and increasing differentiation from the outside towards the inside of the ventricles (Sedmera et al., 2000).

Our results provide further evidence that significant proportions of myocytic aggregates deviate markedly from the tangential plane. At early developmental stages, more than 10% of myocytes within the RV exhibited an angle of intrusion or extrusion that exceed ±45°, while this proportion decreased with gestational age by up to ∼5%. However, the proportion of myocytes within the LV with an angle of intrusion higher than ±45° does not change during gestation and is ∼5%. This suggests that, at early developmental stages (E14-5–E15.5), the RV myocardium appear to be less organised than the LV myocardium. Our values agreed with those reported in previous studies (Lunkenheimer et al., 2006; Schmid et al., 2007; Stephenson et al., 2018). We have also found that the mean angle of intrusion changes from positive values in the base towards negative values in the mid-ventricle, especially from E16.5 onwards, which is consistent with previous studies in humans (Lunkenheimer et al., 2006), rats (Teh et al., 2016), porcine (Schmid et al., 2007) and mice (Garcia-Canadilla et al., 2019).

We have shown that FA gradually increases with developmental age within both ventricles, particularly from E16.5 to E18.5. However, FA in the septum is significantly lower than in the LV and RV. Abdullah et al. reported differences in FA between the RV and LV (including the septum), and he hypothesised it was likely because myocytes in the fetal RV are larger than myocytes in the LV (Abdullah et al., 2016). The increase in FA with development is potentially due to changes in cell size and shape, since myocytes length-to-width ratio increases. Therefore, our results suggest that myocytes in the septum have different shape and size than myocytes in the rest of ventricular walls. Some previous studies in mice have shown that at early developmental stages, myocytes have a more rounded shape, thus they are highly isotropic. Then, from E13.5 gestation, myocytes begin to elongate, hypertrophy and myofibrils become aligned (Hirschy et al., 2006). This process, however, is slow and continues postnatally. A similar process has been observed in the human fetal heart (Mekkaoui et al., 2013; Pervolaraki et al., 2013). However other DT-MRI studies in human fetal hearts have reported that FA decreases with gestational age (Abdullah et al., 2016; Nishitani et al., 2020). These differences among studies could be due to different fixation, sample preparation processes and acquisition settings, that can affect diffusion properties and hence DT-MRI measurements (Agger et al., 2015; Mazumder et al., 2016). In our study, all the samples were prepared, imaged, and quantified following the same procedure. Therefore, we are confident that changes with gestational age in myocyte aggregates orientation and organisation parameters can be attributed to ventricular growth.

We have previously demonstrated that MDI, a parameter that quantifies the similarity in the alignment of myocytes within an ROI, is a good descriptor of myocardial disarray/disorganisation (Garcia-Canadilla et al., 2019; Novo Matos et al., 2020; Planinc et al., 2021). In this work, we have shown that MDI increases gradually with gestational age in both ventricles, indicative of the increasing organisation of the ventricles with development, as illustrated also in Figure 8. However, MDI is higher in LV than in the interventricular septum, suggesting that, in prenatal life, LV myocardium is more organised than septal myocardium.

It is well known that the left and right ventricles are derived from different embryonic origins: the first (LV) and the second or anterior (RV) heart fields (Verzi et al., 2005; Schlüter et al., 2018). Contributions of these hearts fields to the ventricular septum has been a matter of debate, with Verzi et al. (2005) suggesting, for example, that the ventricular septum and proximal portions of the LV are also derived from the anterior/second heart field. The epicardium, and main coronary vasculature are also thought to have distinct embryonic origins. This could help explain why, in our study, the ventricular septum, which aligns with the major interventricular coronaries, shows a different myocardial organisation and growth pattern throughout development compared to the remainder of the ventricular walls. Recently, Nguyen-Truong et al. (2021) showed that the interventricular septum is also biomechanically distinct from the ventricular free walls. More specifically, they found that both sides of the septum were significantly softer with less collagen content than the ventricular walls, supporting the concept that the ventricular septum does not belong to any particular chamber, and should be considered separately since it contributes to both LV and RV function.

In conclusion, the present study provided, for the first time, a comprehensive detailed analysis of ventricular myoarchitecture development in mice. This work provides an important basis for the understanding of the how myoarchitecture develops.

## Data Availability

The original contributions presented in the study are included in the article/Supplementary Material, further inquiries can be directed to the corresponding author.
