# Supplementary material for: Detailed quantification of cardiac ventricular myocardial architecture in the embryonic and fetal mouse heart by application of structure tensor analysis to high resolution episcopic microscopic data
Source: Front Cell Dev Biol. 2022 Nov 16;10:1000684. doi: 10.3389/fcell.2022.1000684 (PMC9709216; doi:10.3389/fcell.2022.1000684)
Supplement: Supplementary file 1 [file DataSheet2.DOCX]

Supplementary Material

# Supplementary Figures


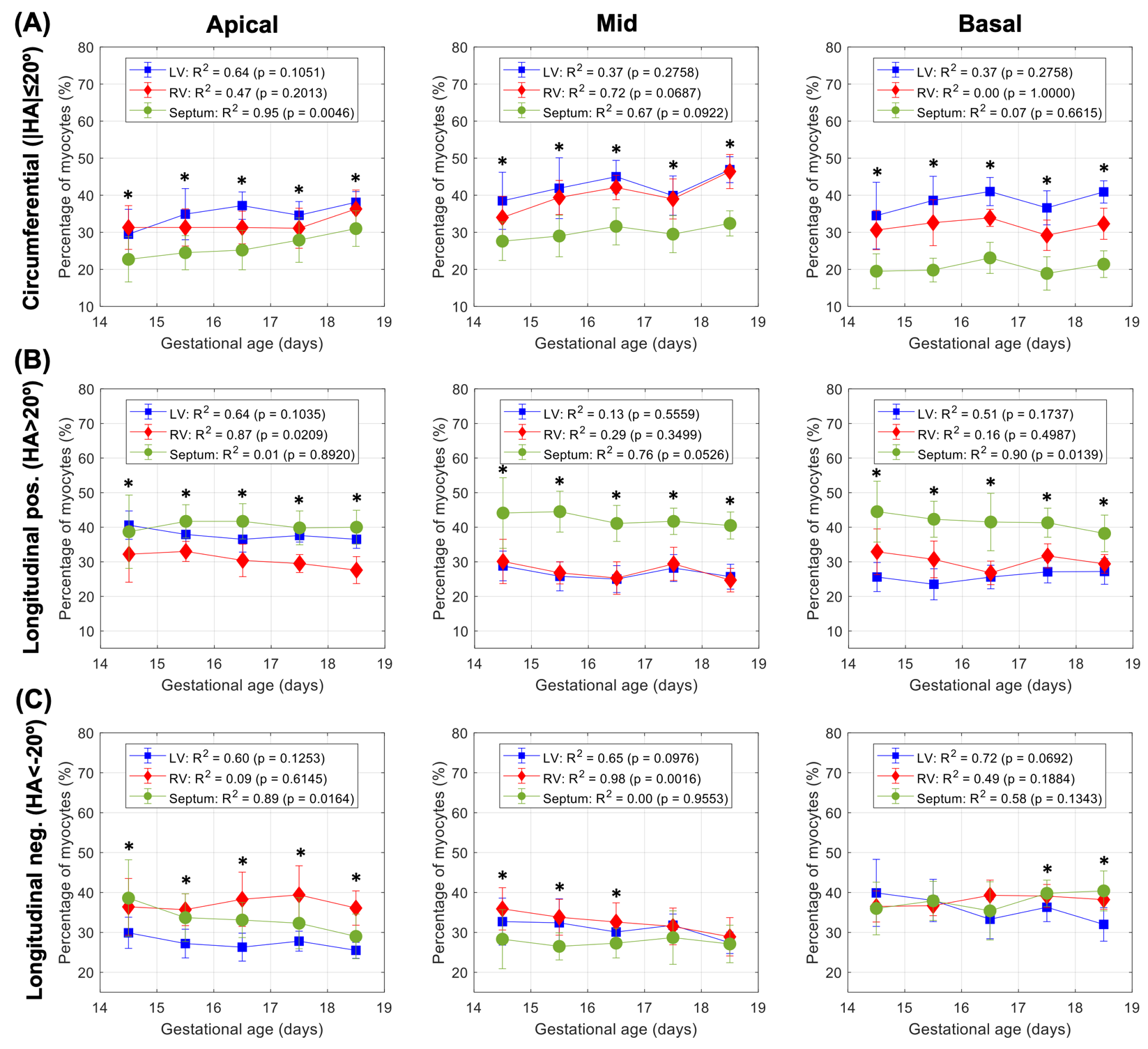


**Supplementary Figure 1**. Percentage of (**A**) circumferentially, (**B**) longitudinally positive and (**C**) longitudinally negative arranged myocytes within the left ventricle (LV, blue squares), right ventricle (RV, red diamonds) and interventricular septum (green circles) for apical, mid-myocardium, and basal slices, plotted as a function of gestational age. Data points are displayed for each gestational age as mean ± one standard deviation. R^2^ values of the linear fitting are also provided. *Significant differences between LV, RV and septum (p<0.05).


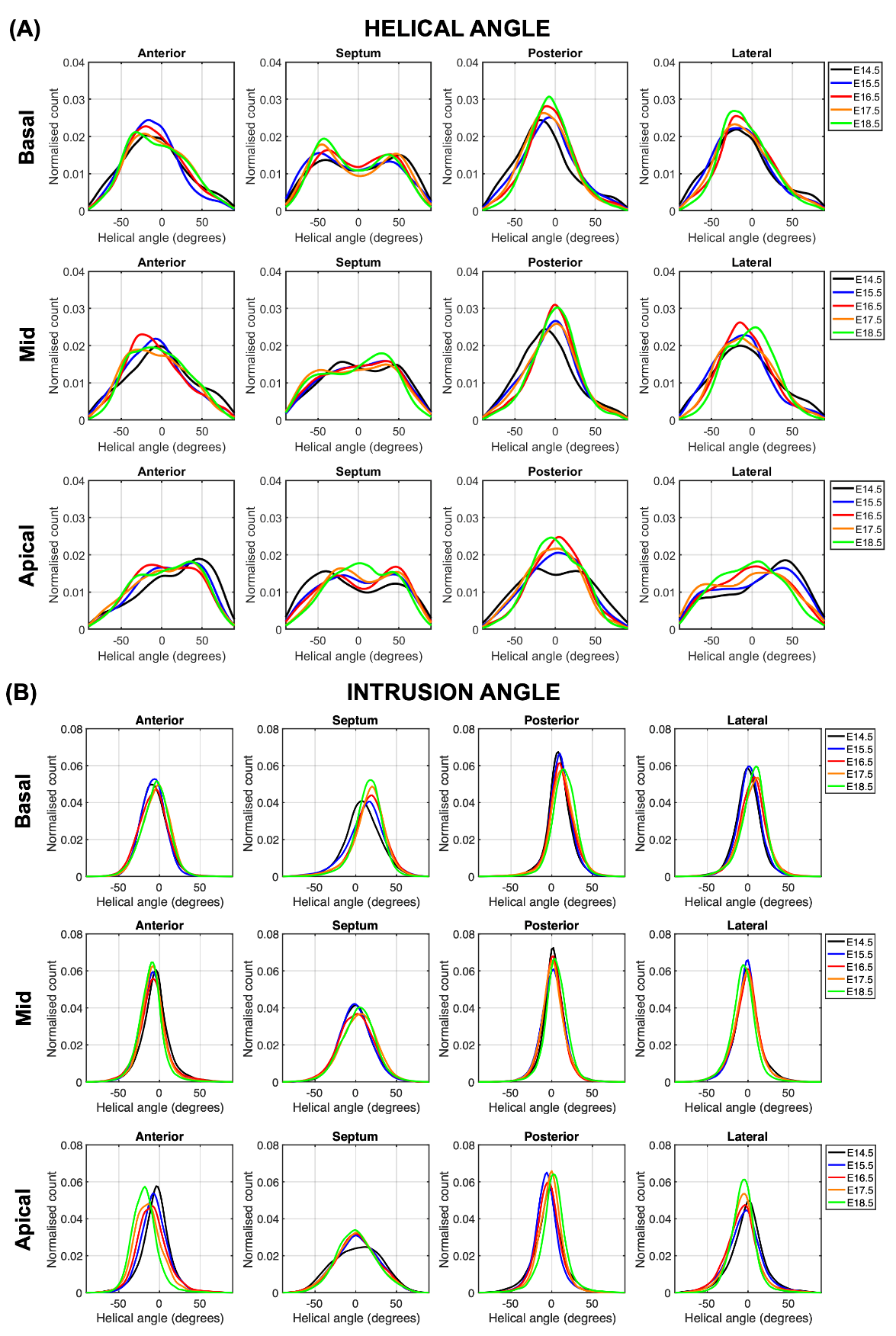


**Supplementary Figure 2**. Segment-specific distributions of **(A)** helical angle (HA) and **(B)** intrusion angle (IA) in the left ventricle (LV), across the four LV walls (anterior, septum, posterior and lateral) and at basal (top), mid (middle) and apical myocardium (bottom) in developing murine hearts at E14.5, E15.5, E16.5, E17.5 and E18.5.


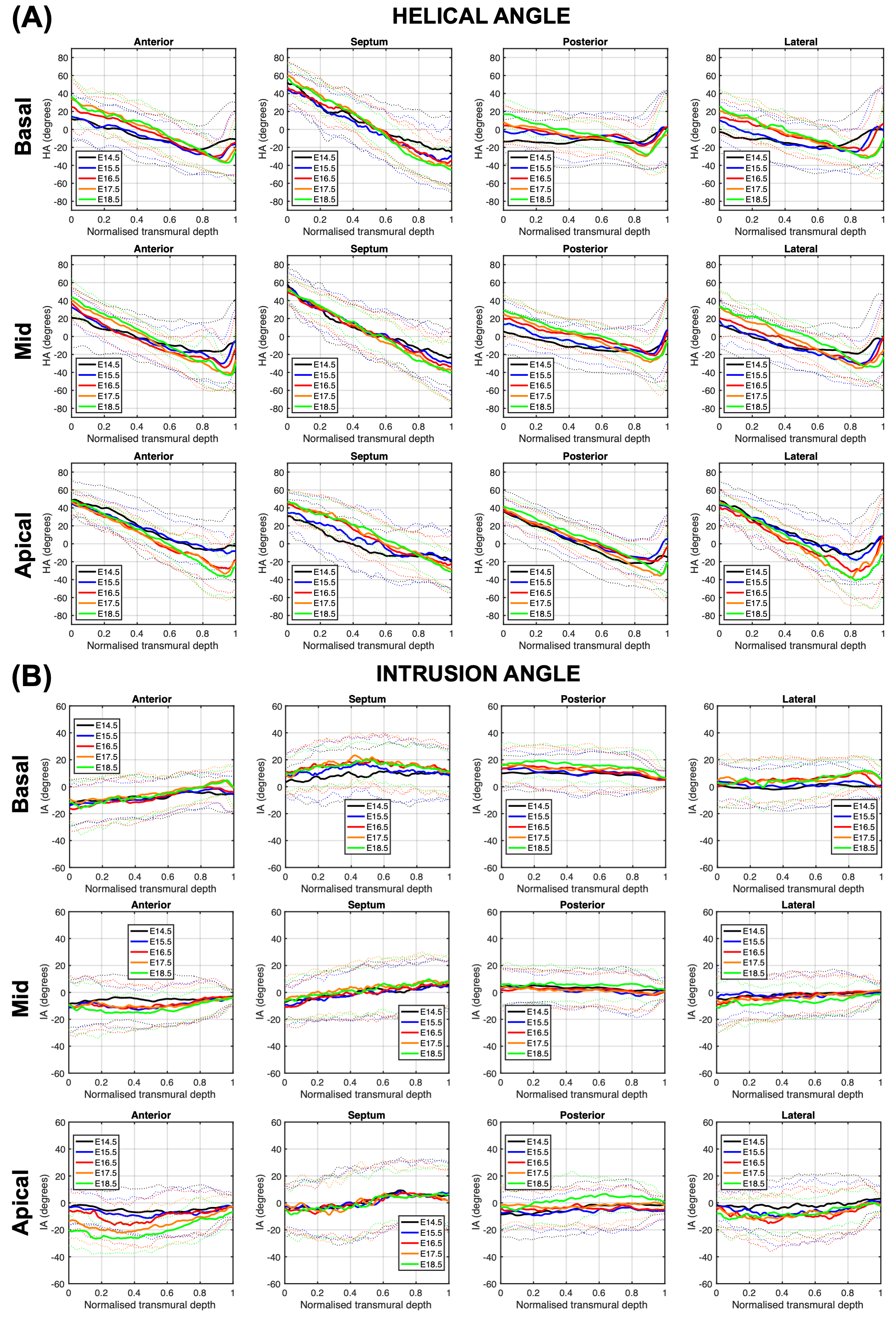


**Supplementary Figure 3**. Left ventricular (LV) transmural profiles of **(A)** helical (HA) and **(B)** intrusion angle (IA) along the normalized transmural depth (0.0; endocardium, 1.0: epicardium or right-side endocardium), across anterior, septal, posterior and lateral walls, at basal (top), mid (middle) and apical myocardium (bottom), in developing murine hearts at E14.5, E15.5, E16.5, E17.5 and E18.5. Solid lines: age-group mean. Dotted lines: ± standard deviation.


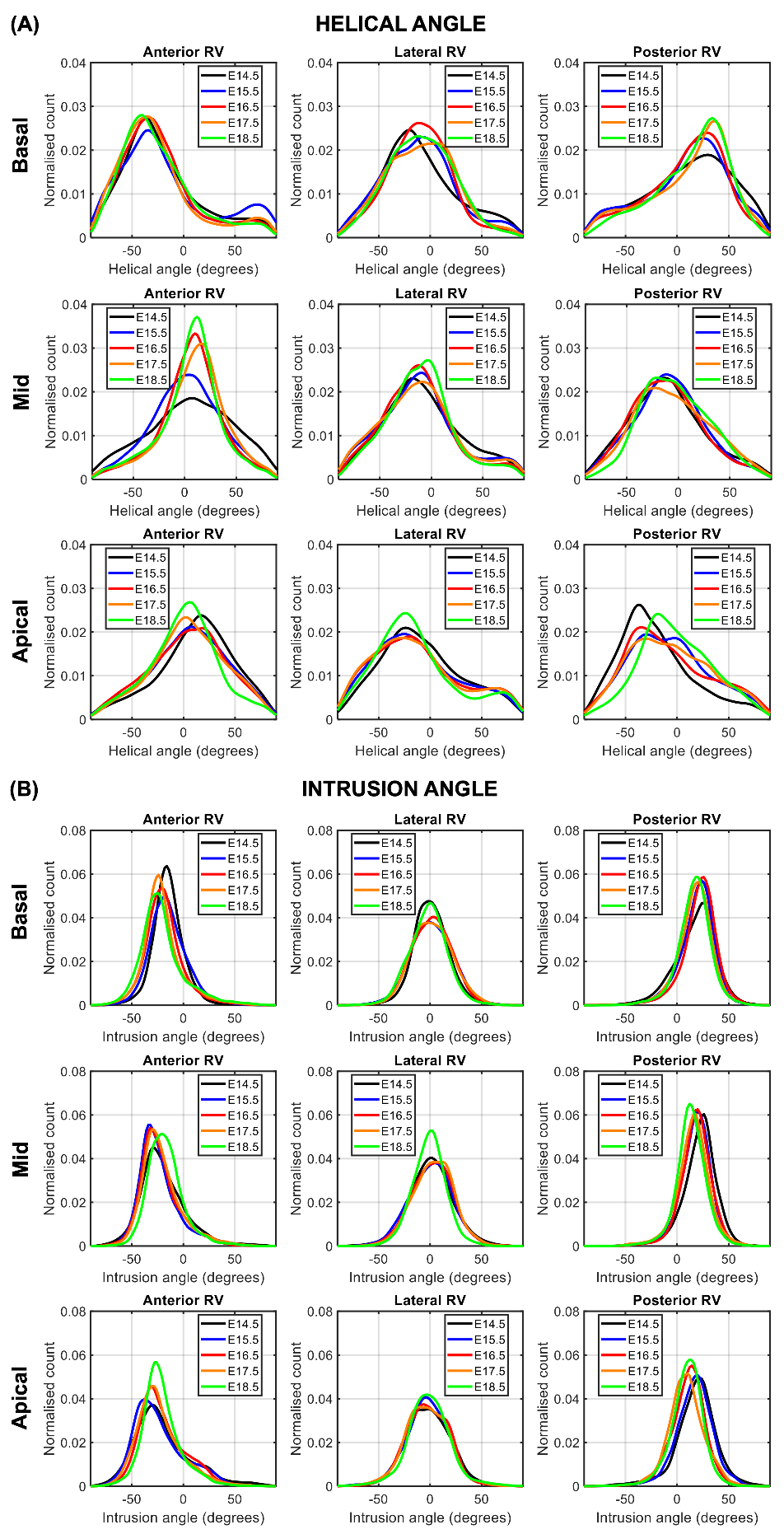


**Supplementary Figure 4**. Segment-specific distributions of **(A)** helical angle (HA) and **(B)** intrusion angle (IA) in the right ventricle (RV), across the three RV walls (anterior, lateral and posterior), at basal (top), mid (middle) and apical myocardium (bottom) in developing murine hearts at E14.5, E15.5, E16.5, E17.5 and E18.5.


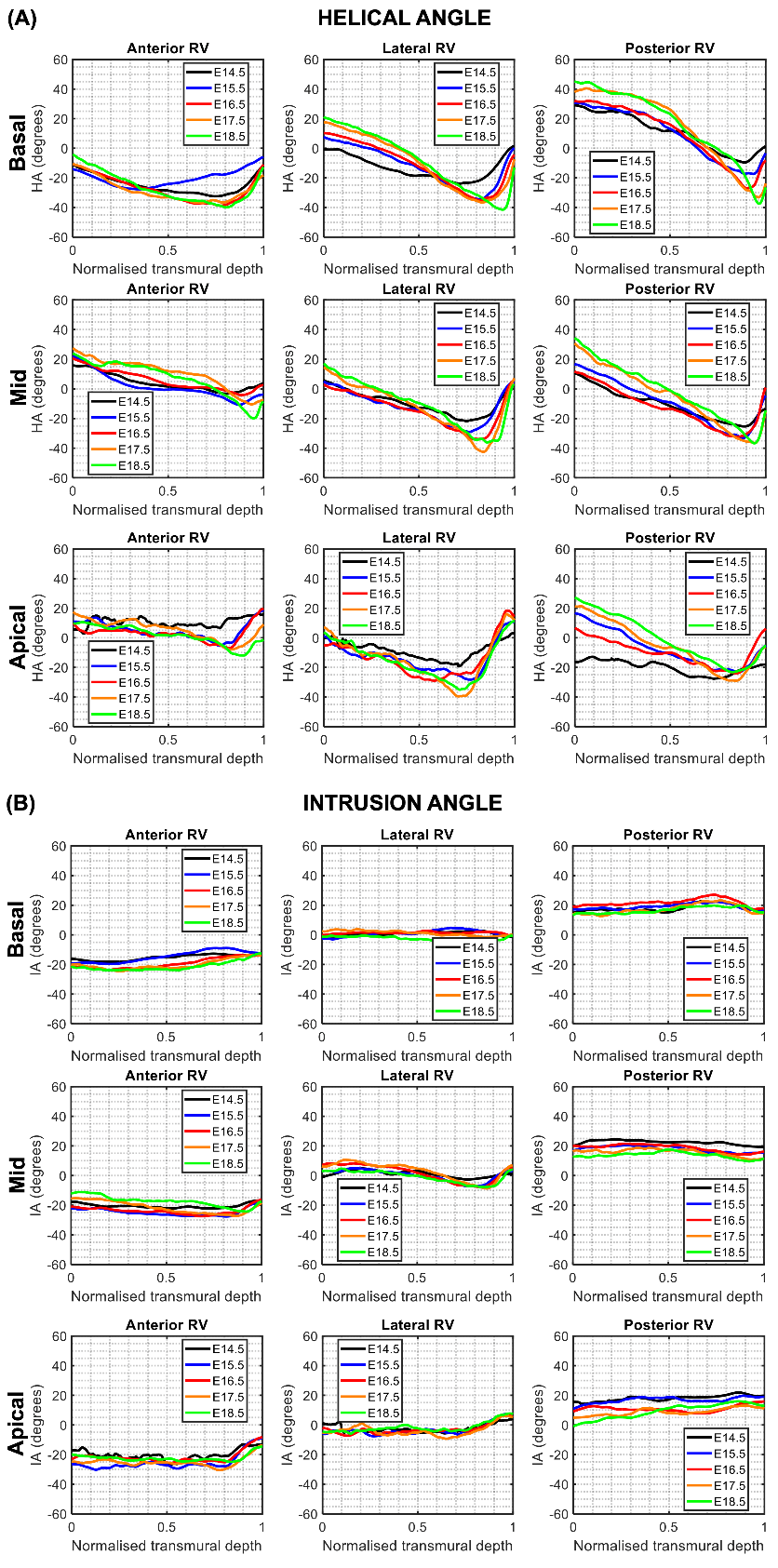


**Supplementary Figure 5**. Right ventricular (RV) transmural profiles of **(A)** helical (HA) and **(B)** intrusion angle (IA) along the normalized transmural depth (0.0; endocardium, 1.0: epicardium), across anterior, lateral and posterior walls, at basal (top), mid (middle) and apical myocardium (bottom), in developing murine hearts at E14.5, E15.5, E16.5, E17.5 and E18.5.


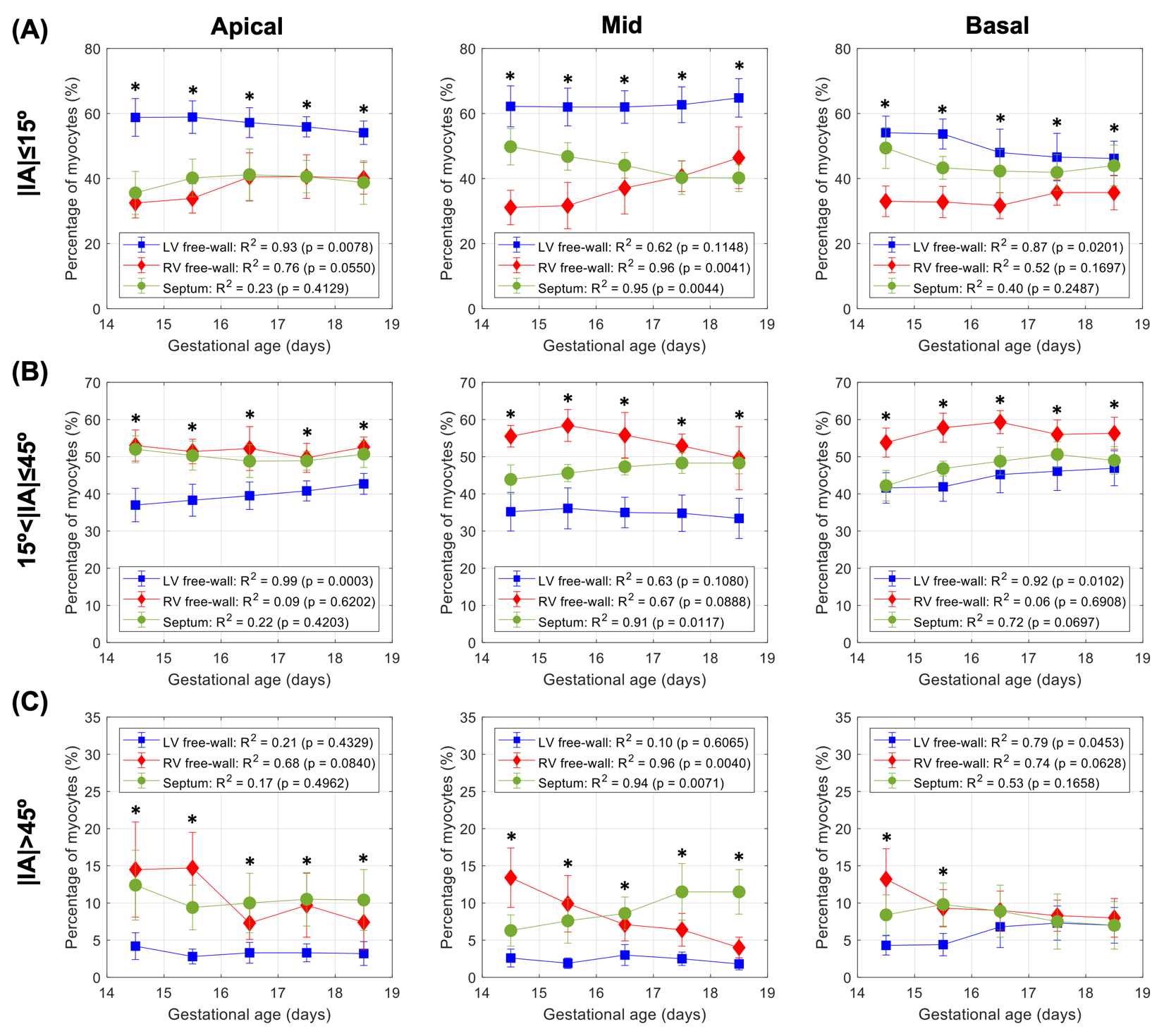


**Supplementary Figure 6**. Percentage of aggregated myocytes with an intrusion angle (IA): (**A**) |IA|≤15º, (**B**) 15º<|IA|≤45º and (**C**) |IA|>45º within the left ventricle (LV, blue squares), right ventricle (RV, red diamonds) and interventricular septum (green circles) for apical, mid-myocardium, and basal slices, plotted as a function of gestational age. Data points are displayed for each gestational age as mean ± one standard deviation. R^2^ values of the linear fitting are also provided. *Significant differences between LV, RV and septum (p<0.05).


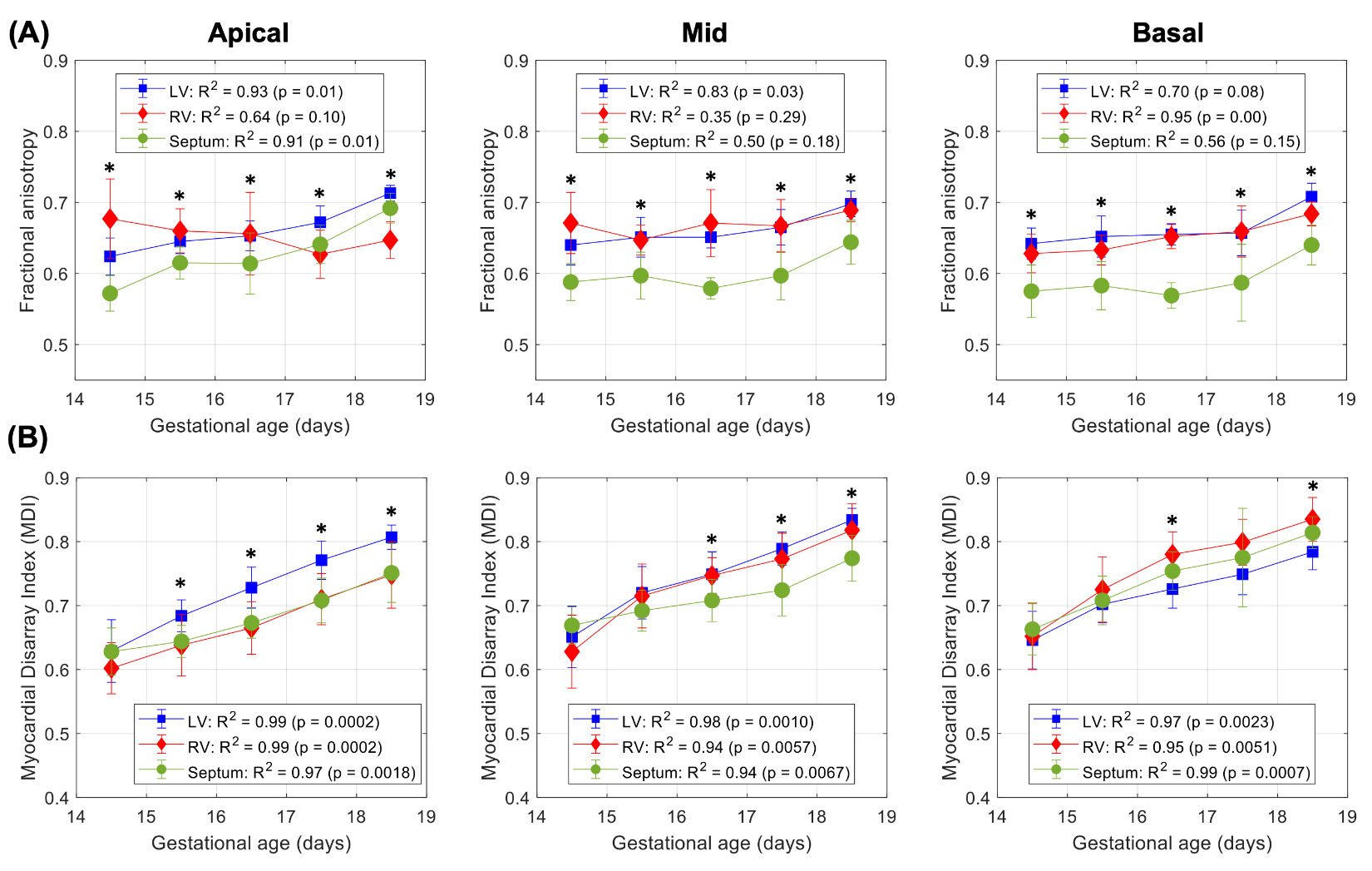


**Supplementary Figure 7**. (**A**) Mean fractional anisotropy (FA) and (**B**) myocardial disarray index (MDI) within the left ventricle (LV, blue squares), right ventricle (RV, red diamonds) and interventricular septum (green circles) for apical, mid-myocardium, and basal slices, plotted as a function of gestational age. Data points are displayed for each gestational age as mean ± one standard deviation. R^2^ values of the linear fitting are also provided. *Significant differences between LV, RV and septum (p<0.05).

**Supplementary Tables**

**Supplementary Table 1.** Parameters of the linear fitting: *y = β_1_·x + β_0_*, of intrusion angle (IA) transmural profile within the different left ventricular walls.

|  | **E14.5** | **E15.5** | **E16.5** | **E17.5** | **E18.5** | **p-value** |
| --- | --- | --- | --- | --- | --- | --- |
| Nº samples | 14 | 14 | 12 | 12 | 14 |  |
| ***Gradient (β_1_) of IA transmural profile (º)*** | | | | | |  |
| Basal Anterior | 7.43 ± 8.78^a^ | 11.82 ± 4.65^ab^ | 17.43 ± 4.82^b^ | 16.33 ± 11.75^ab^ | 18.71 ± 12.25^b^ | 0.010 |
| Basal Septal | 6.06 ± 7.81^a^ | -0.04 ± 11.20^a^ | 2.64 ± 9.54^a^ | 2.98 ± 11.12^a^ | 1.22 ± 12.32^a^ | 0.620 |
| Basal Posterior | -3.72 ± 6.26^a^ | -5.94 ± 4.27^a^ | -10.28 ± 3.95^a^ | -6.39 ± 8.61^a^ | -8.31 ± 6.95^a^ | 0.095 |
| Basal Lateral | 2.07 ± 4.29^ab^ | 0.72 ± 6.19^a^ | 7.05 ± 8.26^ab^ | 6.68 ± 9.18^ab^ | 9.06 ± 6.44^b^ | 0.011 |
| Mid Anterior | 2.33 ± 6.08^a^ | 4.98 ± 6.78^a^ | 7.57 ± 9.41^a^ | 4.18 ± 7.55^a^ | 7.98 ± 7.43^a^ | 0.261 |
| Mid Septal | 11.99 ± 5.40^a^ | 13.95 ± 5.38^a^ | 16.85 ± 9.46^a^ | 12.66 ± 13.27^a^ | 14.33 ± 8.43^a^ | 0.674 |
| Mid Posterior | -4.16 ± 4.91^a^ | -5.16 ± 4.89^a^ | -3.18 ± 3.88^a^ | -4.09 ± 4.27^a^ | -1.63 ± 5.87^a^ | 0.397 |
| Mid Lateral | 4.86 ± 7.27^ab^ | 0.57 ± 8.62^a^ | 10.13 ± 9.43^b^ | 8.06 ± 6.88^ab^ | 7.52 ± 9.42^ab^ | 0.048 |
| Apical Anterior | -1.11 ± 7.01^a^ | 0.02 ± 7.55^a^ | 6.66 ± 8.57^a^ | 15.25 ± 7.08^b^ | 16.50 ± 8.41^b^ | <0.001 |
| Apical Septal | 14.31 ± 9.64^a^ | 13.48 ± 10.15^a^ | 15.18 ± 12.66^a^ | 13.06 ± 11.80^a^ | 16.02 ± 10.38^a^ | 0.956 |
| Apical Posterior | 9.64 ± 7.08^b^ | 3.19 ± 7.71^ab^ | 0.75 ± 7.27^a^ | 2.57 ± 6.61^ab^ | 8.54 ± 5.99^b^ | 0.005 |
| Apical Lateral | 4.94 ± 4.99^a^ | 4.59 ± 11.40^a^ | 9.08 ± 4.64^a^ | 11.79 ± 12.98^a^ | 10.07 ± 8.08^a^ | 0.170 |
| ***Linearity (R^2^) of the linear fitting of IA transmural profile (unitless)*** | | | | | |  |
| Basal Anterior | 0.37 ± 0.27^a^ | 0.54 ± 0.23^a^ | 0.62 ± 0.18^a^ | 0.58 ± 0.26^a^ | 0.62 ± 0.25^a^ | 0.055 |
| Basal Septal | 0.21 ± 0.21^a^ | 0.21 ± 0.22^a^ | 0.19 ± 0.21^a^ | 0.21 ± 0.18^a^ | 0.30 ± 0.24^a^ | 0.652 |
| Basal Posterior | 0.24 ± 0.23^a^ | 0.27 ± 0.21^a^ | 0.43 ± 0.19^a^ | 0.25 ± 0.28^a^ | 0.32 ± 0.26^a^ | 0.256 |
| Basal Lateral | 0.18 ± 0.21^a^ | 0.18 ± 0.15^a^ | 0.27 ± 0.23^a^ | 0.24 ± 0.25^a^ | 0.26 ± 0.20^a^ | 0.692 |
| Mid Anterior | 0.20 ± 0.15^a^ | 0.21 ± 0.25^a^ | 0.25 ± 0.24^a^ | 0.23 ± 0.18^a^ | 0.32 ± 0.28^a^ | 0.675 |
| Mid Septal | 0.43 ± 0.24^a^ | 0.50 ± 0.23^a^ | 0.50 ± 0.29^a^ | 0.44 ± 0.26^a^ | 0.48 ± 0.28^a^ | 0.931 |
| Mid Posterior | 0.28 ± 0.26^a^ | 0.28 ± 0.22^a^ | 0.22 ± 0.23^a^ | 0.21 ± 0.22^a^ | 0.19 ± 0.23^a^ | 0.778 |
| Mid Lateral | 0.27 ± 0.19^a^ | 0.28 ± 0.22^a^ | 0.41 ± 0.27^a^ | 0.30 ± 0.23^a^ | 0.32 ± 0.27^a^ | 0.567 |
| Apical Anterior | 0.15 ± 0.22^a^ | 0.14 ± 0.15^a^ | 0.19 ± 0.17^a^ | 0.43 ± 0.22^b^ | 0.47 ± 0.21^b^ | <0.001 |
| Apical Septal | 0.31 ± 0.16^a^ | 0.36 ± 0.24^a^ | 0.30 ± 0.20^a^ | 0.29 ± 0.13^a^ | 0.38 ± 0.27^a^ | 0.737 |
| Apical Posterior | 0.37 ± 0.25^a^ | 0.14 ± 0.18^a^ | 0.23 ± 0.22^a^ | 0.18 ± 0.21^a^ | 0.31 ± 0.21^a^ | 0.053 |
| Apical Lateral | 0.14 ± 0.16^a^ | 0.23 ± 0.24^a^ | 0.25 ± 0.20^a^ | 0.32 ± 0.28^a^ | 0.30 ± 0.25^a^ | 0.257 |

Superscripts represent non-significant differences between corresponding pairs, at p = 0.05 as analyzed by Tukey’s HSD.

**Supplementary Table 2.** Parameters of the linear fitting: *y = β_1_·x + β_0_*, of intrusion angle (IA) transmural profile within the different right ventricular walls.

|  | **E14.5** | **E15.5** | **E16.5** | **E17.5** | **E18.5** | **p-value** |
| --- | --- | --- | --- | --- | --- | --- |
| Nº samples | 14 | 14 | 12 | 12 | 14 |  |
| ***Gradient (β_1_) of IA transmural profile (º)*** | | | | | |  |
| Basal Anterior | 6.20 ± 3.96^a^ | 12.18 ± 5.26^a^ | 10.71 ± 6.48^a^ | 7.51 ± 8.30^a^ | 8.30 ± 10.15^a^ | 0.206 |
| Basal Lateral | 1.77 ± 4.93^ab^ | 4.54 ± 10.76^a^ | 0.22 ± 6.13^ab^ | -6.44 ± 7.84^b^ | -2.36 ± 13.37^ab^ | 0.043 |
| Basal Posterior | 4.24 ± 9.99^a^ | 2.61 ± 6.91^a^ | 1.58 ± 9.74^a^ | 6.51 ± 7.30^a^ | 5.58 ± 8.56^a^ | 0.591 |
| Mid Anterior | -0.27 ± 11.53^a^ | -1.30 ± 9.48^a^ | -1.47 ± 7.14^ab^ | -13.93 ± 9.87^c^ | -11.37 ± 6.44^bc^ | <0.001 |
| Mid Lateral | -4.55 ± 8.05^a^ | -9.10 ± 7.46^ab^ | -15.21 ± 5.46^b^ | -15.64 ± 4.40^b^ | -10.78 ± 10.28^ab^ | 0.002 |
| Mid Posterior | -3.41 ± 6.97^a^ | -5.48 ± 6.05^a^ | -5.84 ± 7.90^a^ | -4.55 ± 7.16^a^ | -2.55 ± 8.04^a^ | 0.743 |
| Apical Anterior | 3.06 ± 29.85^a^ | 11.71 ± 18.03^a^ | 3.59 ± 14.24^a^ | 0.19 ± 10.87^a^ | 0.59 ± 9.86^a^ | 0.468 |
| Apical Lateral | -3.28 ± 38.87^a^ | 8.47 ± 7.55^a^ | 8.37 ± 9.14^a^ | 2.85 ± 10.21^a^ | 8.47 ± 12.53^a^ | 0.455 |
| Apical Posterior | 4.64 ± 15.59^a^ | 4.33 ± 12.58^a^ | 3.86 ± 6.51^a^ | 9.58 ± 9.10^a^ | 16.03 ± 13.61^a^ | 0.051 |
| ***Linearity (R^2^) of the linear fitting of IA transmural profile (unitless)*** | | | | | |  |
| Basal Anterior | 0.42 ± 0.28^a^ | 0.50 ± 0.27^a^ | 0.46 ± 0.29^a^ | 0.37 ± 0.24^a^ | 0.28 ± 0.34^a^ | 0.319 |
| Basal Lateral | 0.21 ± 0.22^a^ | 0.29 ± 0.32^a^ | 0.31 ± 0.27^a^ | 0.47 ± 0.26^a^ | 0.44 ± 0.29^a^ | 0.105 |
| Basal Posterior | 0.22 ± 0.20^a^ | 0.21 ± 0.20^a^ | 0.28 ± 0.26^a^ | 0.22 ± 0.29^a^ | 0.29 ± 0.31^a^ | 0.898 |
| Mid Anterior | 0.39 ± 0.27^a^ | 0.23 ± 0.30^a^ | 0.17 ± 0.28^a^ | 0.48 ± 0.23^a^ | 0.41 ± 0.29^a^ | 0.035 |
| Mid Lateral | 0.34 ± 0.22^a^ | 0.35 ± 0.27^a^ | 0.49 ± 0.20^a^ | 0.44 ± 0.17^a^ | 0.40 ± 0.28^a^ | 0.508 |
| Mid Posterior | 0.23 ± 0.21^a^ | 0.25 ± 0.20^a^ | 0.34 ± 0.27^a^ | 0.19 ± 0.23^a^ | 0.16 ± 0.25^a^ | 0.362 |
| Apical Anterior | 0.38 ± 0.32^a^ | 0.34 ± 0.24^a^ | 0.30 ± 0.29^a^ | 0.25 ± 0.26^a^ | 0.25 ± 0.25^a^ | 0.680 |
| Apical Lateral | 0.26 ± 0.20^a^ | 0.21 ± 0.20^a^ | 0.25 ± 0.21^a^ | 0.16 ± 0.20^a^ | 0.28 ± 0.28^a^ | 0.671 |
| Apical Posterior | 0.18 ± 0.23^a^ | 0.26 ± 0.30^ab^ | 0.26 ± 0.16^ab^ | 0.34 ± 0.30^ab^ | 0.54 ± 0.29^b^ | 0.009 |

Superscripts represent non-significant differences between corresponding pairs, at p = 0.05 as analyzed by Tukey’s HSD
